# Identification of a Novel Quinvirus in the Family *Betaflexiviridae* That Infects Winter Wheat

**DOI:** 10.3389/fmicb.2021.715545

**Published:** 2021-08-19

**Authors:** Hideki Kondo, Naoto Yoshida, Miki Fujita, Kazuyuki Maruyama, Kiwamu Hyodo, Hiroshi Hisano, Tetsuo Tamada, Ida Bagus Andika, Nobuhiro Suzuki

**Affiliations:** ^1^Institute of Plant Science and Resources (IPSR), Okayama University, Kurashiki, Japan; ^2^Agricultural Research Institute, HOKUREN Federation of Agricultural Cooperatives, Naganuma, Japan; ^3^College of Plant Health and Medicine, Qingdao Agricultural University, Qingdao, China

**Keywords:** *Betaflexiviridae*, quinvirus, bymovirus, yellow mosaic disease, wheat, virome, soil borne, variants

## Abstract

Yellow mosaic disease in winter wheat is usually attributed to the infection by bymoviruses or furoviruses; however, there is still limited information on whether other viral agents are also associated with this disease. To investigate the wheat viromes associated with yellow mosaic disease, we carried out *de novo* RNA sequencing (RNA-seq) analyses of symptomatic and asymptomatic wheat-leaf samples obtained from a field in Hokkaido, Japan, in 2018 and 2019. The analyses revealed the infection by a novel betaflexivirus, which tentatively named wheat virus Q (WVQ), together with wheat yellow mosaic virus (WYMV, a bymovirus) and northern cereal mosaic virus (a cytorhabdovirus). Basic local alignment search tool (BLAST) analyses showed that the WVQ strains (of which there are at least three) were related to the members of the genus *Foveavirus* in the subfamily *Quinvirinae* (family *Betaflexiviridae*). In the phylogenetic tree, they form a clade distant from that of the foveaviruses, suggesting that WVQ is a member of a novel genus in the *Quinvirinae*. Laboratory tests confirmed that WVQ, like WYMV, is potentially transmitted through the soil to wheat plants. WVQ was also found to infect rye plants grown in the same field. Moreover, WVQ-derived small interfering RNAs accumulated in the infected wheat plants, indicating that WVQ infection induces antiviral RNA silencing responses. Given its common coexistence with WYMV, the impact of WVQ infection on yellow mosaic disease in the field warrants detailed investigation.

## Introduction

Wheat (*Triticum aestivum* L.) belongs to the grass family Poaceae and is one of the most important crops worldwide. Several diseases caused by vector-borne viruses have significant impacts on cereal crops, including wheat and barley (Lapierre and Hariri, 2008; Ordon et al., 2009; Jiang et al., 2020). Among these, soil-borne viruses, bymoviruses (family *Potyviridae*, phylum *Pisuviricota*), and furoviruses (family *Virgaviridae*, phylum *Kitrinoviricota*) are known as disease agents for yellow mosaic disease in wheat and barley and have been distributed widely in wheat/barley-growing regions of many countries (Kanyuka et al., 2003; Kuhne, 2009; Jiang et al., 2020). Yellow mosaic is one of the important diseases in winter barley and wheat in Japan and China (Kashiwazaki et al., 1989; Chen et al., 1999; Han et al., 2000; Nishigawa et al., 2008; Ohki et al., 2014).

Wheat yellow mosaic virus (WYMV, a bymovirus) is the causal agent of the wheat yellow mosaic disease and is vectored by the obligate root-inhabiting *Polymyxa graminis* (a protist in the plasmodiophorid group; Inouye, 1969; Tamada and Kondo, 2013). WYMV has a bipartite plus-strand RNA genome (4.6 and 3.6 kb, respectively) that encodes large polyproteins (Namba et al., 1998; Wylie et al., 2017). WYMV and other bymoviruses require cool temperatures for multiplication, and their symptoms on infected leaves are masked when the average temperature exceeds 20°C (Cadle-Davidson and Bergstrom, 2004; Jiang et al., 2020). WYMV is widespread in the wheat production areas of Japan and China with the range of estimated yield losses due to the disease being 20–40% (Han et al., 2000; Zhang et al., 2011; Ohki et al., 2014). A closely related bymovirus – wheat spindle streak mosaic virus, which causes a similar yellow mosaic disease on winter wheat – is distributed mainly in North America and European countries (Clover and Henry, 1999; Jiang et al., 2020).

More than 57 viruses that infect wheat or barley have been identified using conventional diagnostic methods (Lapierre and Hariri, 2008). In recent years, the RNA sequencing (RNA-seq) analyses of field-grown crops, using next-generation sequencing (NGS) techniques, have enabled a more-detailed view on viral communities in the agroecosystem, often leading to new virus discoveries (Roossinck et al., 2015). Several studies have investigated the virome of wheat and barley plants using deep RNA-seq (Jo et al., 2018; Golyaev et al., 2019; Albrecht et al., 2020; Hodge et al., 2020; Singh et al., 2020), and some have identified the novel or uncharacterized wheat-infecting viruses, such as wheat stripe mosaic virus (a tentative benyvirus in the *Benyviridae*), wheat yellow striate virus (an alphanucleorhabdovirus in the family *Rhabdoviridae*), and European wheat striate mosaic virus (a putative tenuivirus in the family *Phenuiviridae*; Liu et al., 2018; Valente et al., 2019; Somera et al., 2020). Nevertheless, there is still very limited information concerning virome or viral agents in the wheat plants that are associated with yellow mosaic disease.

The members of the family *Betaflexiviridae* (order *Tymovirales*, phylum *Kitrinoviricota*) form two phylogenetically separate clades that are assigned to two subfamilies – *Trivirinae* and *Quinvirinae* (Adams et al., 2016). The subfamily *Quinvirinae* contains three genera – *Carlavirus*, *Foveavirus* and, *Robigovirus* – and three floating species, including *Banana mild mosaic virus* (recently proposed genus “Banmivirus”), *Banana virus X*, and *Sugarcane striate mosaic associated virus* (proposed genus “Sustrivirus”; Candresse et al., 2021). Quinviruses have a plus-sense RNA genome (5.8–9.0 kb; 5' capped and 3' polyadenylated ends) with six open reading frames (ORFs) for carlaviruses or five ORFs for the other two genera and floating species, with some exceptions (Caglayan et al., 2019; Zheng et al., 2020). Members of the *Quinvirinae* encode the so-called triple-gene block (TGB) for cell-to-cell movement in the leaf epidermis, and are clearly differentiated from the members of the subfamily *Trivirinae*, whose genomes encode a single movement protein (the 30K superfamily). Most quinviruses belonging to the genera *Foveavirus* (eight species) and *Robigovirus* (five species) naturally infect fruit trees, such as apple, cherry, and grapevine, while the members of the genus *Carlavirus* (53 species) infect various dicotyledonous plants (Ryu and Song, 2021; Yoshikawa and Yaegashi, 2021). Recently, several foveavirus candidates have also been reported from diseased fruit trees and garlic (*Allium sativum*) plants (Park et al., 2019; Gazel et al., 2020; Reynard et al., 2020; Yaegashi et al., 2020; Zheng et al., 2020; Luo et al., 2021). Some carlaviruses are transmitted by hemipteran insects, such as aphids and whiteflies, in non-persistent manners; while there have been no reports on the vectors of foveaviruses or robigoviruses, which raises speculation about their mechanical- or graft-transmission in the field (Ryu and Song, 2021). A quinvirus (sugarcane striate mosaic associated virus, SCSMaV) is apparently soil transmissible *via* an unknown mechanism (Choi et al., 1999; Thompson and Randles, 2001).

In the current study, a *de novo* meta-RNA-seq approach was used to investigate the virome of wheat plants sampled from a field in Hokkaido, Japan. We discovered three strains of a novel quinvirus tentatively named wheat virus Q (WVQ) that often co-infected wheat with a few known viruses. We also found that WVQ could be transmitted *via* soil and occurred at least 4 consecutive years in the field, raising the concerns about its potential impact on wheat production.

## Materials and Methods

### Collection of Plant Materials

The plant materials used in this study were sampled at an experimental field in the HOKUREN Agriculture Research Institute, Naganuma, Hokkaido, Japan (43.3°N) in May and June of 2018 and 2019 (a total of 14 wheat samples or pools; Table 1). The leaves of wheat plants showing yellow mosaic and asymptomatic leaves, including from the cultivars “Kitahonami” (KTH) in 2018−2019, and “Kitanokaori” (KTN) and “Yumechikara” (YM, a WYMV resistant cultivar; Kojima et al., 2015) in 2019, were collected (Supplementary Figure S1 and data not shown). The wheat plants collected in 2019 were grown on plots with or without the fungicide fluazinam (Flu; Ishihara Sangyo Kaisha, LTD.) soil treatment. Wheat (KTH) and rye (*Secale cereale* cv. Fuyumidori) samples (leaves and roots) were also collected from the same Naganuma field in 2020 and 2021 (Table 1). The collected plant materials were stored at −80°C until their analysis.

**Table 1:** List of field-collected samples used in this study and the viruses identified by RNA-seq.

No.	Sample name^1^	Wheat cultivar or plant name	Collected date	NGS group^2^	Virus^3^
2018					
1.	KTH-18-1	cv. Kitahonami	15 May	poo1-18si	W/Q
2	KTH-18-2	cv. Kitahonami	15 May	poo1-18si	W/Q
3.	KTH-18-3	cv. Kitahonami	15 May	NA	W/Q
4.	KTH-18-4	cv. Kitahonami	15 May	NA	W/Q
5.	KTH-18-5	cv. Kitahonami	15 May	NA	W/Q
6.	KTH-18-6 (1+2)	cv. Kitahonami	5 June	pool-18L	Q
7.	KTH-18-7 (3+4)	cv. Kitahonami	5 June	pool-18L	W/N/Q^*^
8.	KTH-18-8 (5+6)	cv. Kitahonami	5 June	pool-18L	W/Q^*^
2019					
9.	KTH-19-1	cv. Kitahonami	18 June	NA	Q
10.	KTH-19-2 (Flu)^4^	cv. Kitahonami	18 June	pool-19L	W/Q
11.	KTN-19-3	cv. Kitahonami	18 June	NA	Q
12.	KTN-19-4 (Flu)^4^	cv. Kitahonami	18 June	pool-19L	(Q)
13.	YM-19-5	cv. Yumechikara	18 June	NA	Q
14.	YM-19-6 (Flu)^4^	cv. Yumechikara	18 June	pool-19L	Q
2020					
15.	KTH-20-1	cv. Kitahonami	11 May	NA	W/Q
16.	Rye-20-2	Rye (*Secale cereale*)	11 May	NA	(W)/Q^*^
2021					
17.	KTH-21-1	cv. Kitahonami	19 April	NA	W/Q
18.	Rye-21-2	Rye (*Secale cereale*)	19 April	NA	(W)/Q^*^

1 All plant materials were taken from an experimental field at HOKUREN Agricultural Research Institute, Naganuma, Japan (43.3°N).

2 Total RNA fractions from the leaf samples were pooled according to the year of collection (pool-18L and pool-19L). Both pooled samples were used for transcriptomic sequencing, while poo1-18si was used for small RNA sequencing (RNA-seq). NA, not applicable.

**Figure 1:**
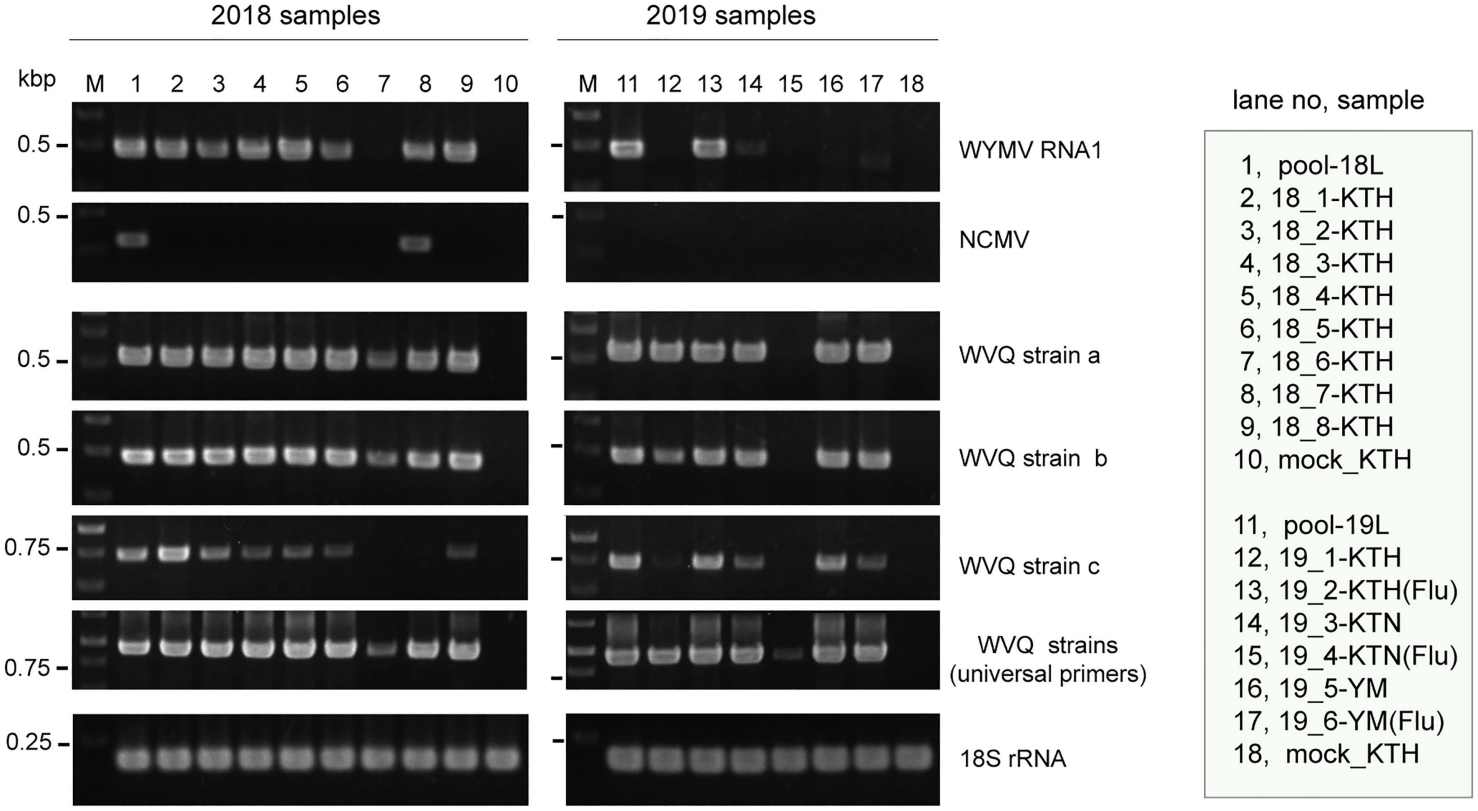
Reverse transcriptase PCR (RT-PCR) detection of viruses in wheat leaf RNA samples. Sample names are shown in the right-hand box. Lane 1 (pool-18L) and lane 11 (pool-19L) are two pooled RNA samples subjected to NGS analysis. Lanes 10 and 18 are RNA samples of mock-inoculated plants. KTH, cv. Kitahonami; KTN, cv. Kitanokaori; YM, cv. Yumechikara; and Flu, fluazinam treatment to the soil. The wheat 18S ribosomal RNA (rRNA) gene was used as an endogenous RNA reference. The primers used in the RT-PCR analyses are listed in Supplementary Table S1. Selected PCR products were sequenced to confirm the amplification of the virus targets. The PCR products were analyzed in 1.5% agarose gel electrophoresis. The gel was stained with ethidium bromide and viewed under UV light.

3 The virus infection was assayed *via* RT-PCR (see the results in Figures 1, 6). W, wheat yellow mosaic virus (WYMV); N, northern cereal mosaic virus (NCMV); and Q, wheat virus Q (WVQ, a novel quinvirus). Parentheses indicate faint PCR products. Asterisks indicate strain c was undetected.

4 Sampled from an experimental plot treated with fluazinam, which is a broad spectrum fungicide.

### *De novo* RNA-Seq Analysis

Meta-RNA-seq analysis was basically conducted as has been described previously (Kondo et al., 2016; Lin et al., 2019). The total RNA from each wheat-leaf sample was extracted using NucleoSpin RNA Plant and Fungi Kit (Macherey and Nagel, Düren, Germany), following the manufacturer’s instructions. The obtained RNA fractions from the 2018 (three samples: KTH-18-6, −7, and −8) and 2019 (three samples: KTH-19-2, −4, and −6) samples were pooled into two groups based on the collection year – pool-18L (total 15.8 μg; RNA integrity number, RIN = 7.9) and pool-19L (total 7.8 μg; RIN = 7.7), respectively (Table 1). The two sample pools were subjected to ribosomal RNA (rRNA) depletion using a Ribo-Zero kit (Illumina, San Diego, CA, United States), and were subsequently used for the synthesis of a cDNA library using TruSeq Stranded Total RNA LT Sample Prep Kit (Plant; Illumina). The two cDNA libraries were then subjected to RNA-seq using the Illumina HiSeq 2000 platform (Illumina, 100-bp pair-end reads) performed by Macrogen Corp. Japan (Tokyo, Japan). After RNA-seq, the sequence reads were trimmed and *de novo* assembled using the CLC Genomics Workbench version 11 (CLC Bio-Qiagen, Aarhus, Denmark) using default parameters (minimum contig length = 200; mismatch cost = 2; length fraction = 0.5; similarity fraction = 0.8). Subsequently, assembled contigs were used as queries for basic local alignment search tool (BLAST) analyses (all contigs or contigs larger than 1.0 kb were subjected to BLAST-N or BLAST-X search, respectively) against the viral genome reference sequence (RefSeq) dataset of the National Center for Biotechnology Information (NCBI^1^; E-value cut-off set >0.05). Quinvirus-like sequences from public datasets were also used as queries for BLAST searches against assembled contigs generated in this study to detect further virus-related sequences. The sequence reads (or assembled contigs) were mapped to the assembled virus or virus-like contigs using the Read Mapping algorithm using default or more stringent mapping parameters (match score = 1; mismatch cost = 2; length fraction = 0.5 or 0.9; similarity fraction = 0.8 or 0.95) in the CLC Genomics Workbench.

### RNA Extraction and RT-PCR

The total RNA was extracted from the plant materials using RNAiso Plus Reagent (TaKaRa Biotech. Co., Shiga, Japan) according to the manufacturer’s instructions. The cDNA was synthesized using Moloney-murine leukemia virus (MMLV) reverse transcriptase (Thermo Fisher Scientific, Waltham, MA, United States) with random primers [nonadeoxyribonucleotide mixture; pd. (N)9; TaKaRa Bio], and was then used as a template for PCR amplification with QuickTaq (Toyobo, Osaka, Japan). The conditions of PCR were as follows: 94°C for 2 min; then 30 or 35 cycles of 94°C for 10 s, 53°C or 57°C for 30 s, and 72°C for 1 or 2 min; and 72°C for 10 min. Alternatively, the RNA samples were subjected to one-step reverse transcriptase PCR (RT-PCR) analysis by using PrimeScript One-Step RT-PCR Kit Ver. 2 (TaKaRa), following the manufacturer’s instructions. The 5' and 3' rapid amplification of cDNA ends (5' and 3' RACE) analyses were conducted using the 5'-Full RACE Core Set and 3'-Full RACE Core Set (TaKaRa Bio), respectively, with virus-specific primers (Kondo et al., 2006; Supplementary Table S1). The wheat 18S rRNA gene was used as the reference target of an endogenous gene for the RT-PCR (Jarošová and Kundu, 2010). The primers used in the RT-PCR analyses are listed in Supplementary Table S1 and are available upon request. The selected PCR products were purified and subjected to Sanger sequencing to confirm their nucleotide sequences. For 5' and 3' RACE analyses, the obtained DNA products were ligated into the pGEM-T easy PCR cloning vector (Promega) and transformed into *Escherichia coli* strain DH5 alpha (TaKaRa Bio), and then plasmids were subjected to DNA sequencing.

### Sequence Analysis and Database Search

Sequence data were analyzed using EnzymeX ver. 3.3.3^2^ and 4peaks v1.8.^3^ BLAST or reverse BLAST searches were conducted using the GenBank database through the NCBI web site running on the non-redundant (nr) DNA and protein databases from the NCBI (nucleotide collection – nr/nt; transcriptome shotgun assembly – TSA; and expression sequence tag – EST; see Footnote 1). Sequence identities were also calculated using BLAST program available from NCBI (BLAST-N suite-2 sequence program; see Footnote 1). The conserved domains were searched using the NCBI conserved domain database.^4^ Pairwise sequence comparisons (PASCs) were made using the Sequence Demarcation Tool (SDT) ver. 1.2 (Muhire et al., 2014). A genome-based web tool for virus classification (pairwise sequence comparison – PASC) was used for the novel virus sequence analysis^5^ (Bao et al., 2014).

### Phylogenetic Analysis

For the phylogenetic analyses, maximum-likelihood (ML) tree was constructed, as described in Kondo et al. (2019, 2020), with minor modifications. Multiple amino-acid sequence alignments were generated by using MAFFT online ver. 7^6^ (Katoh and Standley, 2013), with poorly aligned sites being removed using Gblocks online version 0.91b^7^ (Talavera and Castresana, 2007). Phylogenetic trees were then constructed using the PhyML 3.0 online program^8^ (Guindon et al., 2010; Lefort et al., 2017). Neighbor-joining (NJ) trees (Saitou and Nei, 1987) were constructed based on the MAFFT alignments masked with Gblocks. The reliability of the branches was obtained from 100 bootstrap replicates. The trees were refined using FigTree ver. 1.3.1 software.^9^

### Small RNA-Seq

Small RNA deep sequencing was performed as described by Shahi et al. (2019). Two total RNA samples from 2018 (KTH-18-1 and −2) were mixed together (total 14.6 μg; RIN = 7.8) and subjected to small RNA seq (Table 1). After cDNA library preparation using a TruSeq Small RNA Library Prep Kit (Illumina), deep RNA-seq was conducted using an Illumina HiSeq 2500 (Illumina, 150-bp single-end reads) by Macrogen Corp., Japan. The raw sequence reads (total read number, 46,026,536; total read bases, 6,950-Mb) were trimmed of adapters and filtered for low-quality sequences and size range (15 to 32 nt, in length) using the CLC Genomic Workbench. The clean reads were subsequently mapped into the virus genomes. The virus-derived small RNA reads were used for further analysis using the program MISIS-2 (Seguin et al., 2016).

### Virus Transmission Tests

For soil transmission, the soils that were collected from the field with WYMV-infected wheat plants (KTH-20-1) were used as a virus inoculation source. Commercial culture soil (Nihon Hiryo Co., Ltd., Tokyo, Japan) was also used for non-infested healthy soils. Wheat seeds (cv. “Kitahonami”) were sown in plastic pots (7.5 cm in diameter) containing a mixture of the infested soil and the culture soil, and were grown in a growth cabinet (around 16°C, 12 h light/12 h dark) or in a greenhouse (non-controlled temperature conditions, around 15–25°C or occasionally slightly higher). At different periods after sowing, total RNA extracted from the roots of the plants was subjected to RT-PCR.

## Results

### Identification of Virus-Like Sequences From the Wheat-Leaf RNA-Seq Analysis

RNA sequencing analysis of the two pooled, rRNA-depleted RNA preparations of wheat-leaf samples (Table 1; Supplementary Figure S1) resulted in totals of 55,585,610 (5,614 Mb; pool-18L) and 64,002,754 (6,464 Mb; pool-19L) raw reads, respectively. *De novo* assembly using the CLC Genomics Workbench yielded 124,777 (pool-18L) and 131,004 (pool-19L) sequence contigs. These assembled contigs were then subjected to local BLAST-N (all contigs as queries) and BLAST-X (contigs larger than 1.0 kb as queries) analysis against the viral RefSeq collection. At least, 86 contigs (44 from pool-18L and 42 from pool-19L, ranging from 230 to 8,590 nt) were indicated as candidates for virus-derived sequences. The 41 contigs had a sequence similarity to WYMV (a bymovirus; 11 contigs), northern cereal mosaic virus (NCMV, a plant rhabdovirus; 2 contigs), and betaflexiviruses (at least 28 contigs; Supplementary Table S2). The remaining 43 contigs were related to fungal viruses of the families *Narnaviridae*, *Botyourmiaviridae*, and *Mymonaviridae* and some others (data not shown). Approximately, 1.1% of the reads (636,069 reads) in pool-18L and 0.9% of the reads (550,442 reads) in pool-19 were assigned to virus-related reads in each library. Almost all the virus-associated reads were derived from plant viruses – 43.5% (515,962 reads) for WYMV, 0.4% (4,216 reads) for NCMV, and 53.3% (632,472 reads; several unassembled reads related to a betaflexivirus strain might also be presented in the two pools, see below) for betaflexiviruses, with 2.9% (33,861 reads) representing others, likely derived from fungal-associated viruses (Supplementary Table S2 and data not shown).

### Characterization of Two Known Wheat RNA Viruses

We obtained at least 11 contigs of WYMV RNA segments from the pool-18L and pool-19L libraries. Among these, two contigs (Wh18L_c88 and Wh19L_c362) were nearly complete sequences of the WYMV RNA1 segment, while the RNA2 coding-complete sequence was generated using partially overlapping contigs in each dataset (Wh18L_c38/1970/1462 and Wh19L_c740/2865/2864, respectively; Supplementary Figure S2A and Supplementary Table S2). The WYMV sequences obtained from both libraries were almost identical, and the sequences were confirmed by mapping the reads (the representative contig sequences of RNA1 and RNA2 have been deposited in DDBJ under Accession Nos., LC63269 and LC63270, respectively; Table 2). The WYMV segments showed their highest sequence identities with a Japanese isolate Morioka (Accession No., AB627810) for RNA1 (99.6% nucleotide sequence identity) and a Chineses isolate (Shandong, KY354407) for RNA2 (99.0% nucleotide sequence identity).

**Table 2:** Annotated virus contigs from the wheat-leaf RNA-seq analyses.

Virus or tentative virus name	Contig or concatenated name	Size (nt)	Mapped reads no. pool-18/pool-19^1^	Accession no.
WYMV RNA1	Wh19L_c362	7,635^2^	199,947/111,012	LC632069
WYMV RNA2	Wh19L_c740/2865/2864	3,643^2^	142,064/65,799	LC632070
NCMV	Wh18L_c253/20097	13,222^2^	4,186/0	LC632071
WVQ strain a		8,412^3^	166,410/329,116	LC632066
WVQ strain b		8,411^3^	178,802/70,997	LC632067
WVQ strain c		(~725)^4^	(12,531)/(14,320)	LC632068

1 The data were obtained by the Read Mapping algorithm with following parameters: match score = 1; mismatch cost = 2; length fraction = 0.9; and similarity fraction = 0.95.

2 See Supplementary Figures S2A,B and Supplementary Table S2 for the details of the contigs in each virus. Both WYMV and NCMV sequences were not included their terminal regions.

**Figure 2:**
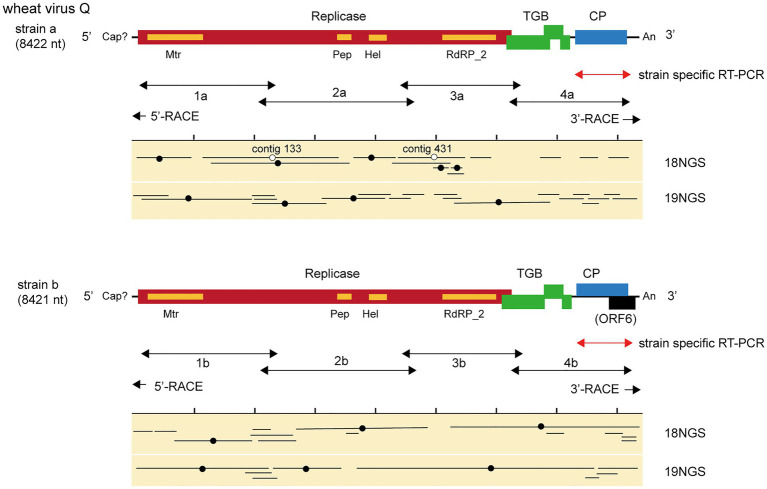
Virus sequences of novel quinvirus (wheat virus Q, WVQ) strains identified from the RNA sequencing (RNA-seq) of wheat leaves. The black lines in the genomic RNA show ORFs. The conserved domains in the WVQ replicase are shown (Mtr, methyltransferase; Pep, Peptidase_C23 superfamily; HEL, RNA helicase; and RdRp_2, RNA-dependent RNA polymerase). The entire genomic sequence of the WVQ strains (a and b) was obtained from the overlapping RT-PCR products and the 5ꞌ and 3ꞌ RACE products (see the double-arrowed or arrowed lines below each schematic genome of the WVQ strains for RT-PCR target regions). A genomic region of WVQ, as indicated by the double-arrowed red lines, was used for strain-specific detection by RT-PCR in Figures 1, 6. The primers used in the RT-PCR and RACE analyses are listed in Supplementary Table S1. Virus-sequence contigs from two separate total RNA pools of wheat leaf samples (18NGS, pool-18L; 19NGS: pool-19L) are shown. The lines marked with a black filled-circle are the contigs listed in Supplementary Table S2, and they were used for constructions of the WVQ genomic sequences. Some unlisted contigs in Supplementary Table S2 were also used for the construction of strain a sequence. The lines marked with an open circle were potentially derived from the variant(s) of WVQ strain a that have sequences slightly different from the reference strain a. The read depth coverage throughout the WVQ genomes (strains a and b) is presented in Supplementary Figure S2C.

3 The entire genomic regions of WVQ strains a and b were determined using RT-PCR and RACE (see Figure 2). The GG (strain a) or G (strain b) nucleotides in the majority of the 5' RACE clones are likely derived from a 5ꞌ-end cap structure.

4 A partial genomic sequence of the third strain c of WVQ (see Supplementary Figure S2C) was also used for map-read analysis.

To verify the presence of WYMV in the RNA samples used for the meta-RNA-seq analyses (Table 1), we performed RT-PCR, using the specific primer sets for RNA1 sequences (Supplementary Table S1). The typical yellow mosaic symptoms on the leaves of the cultivar “Kitahonami” samples are shown in Supplementary Figure S1. WYMV was detected in most of the 2018 “Kitahonami” samples, while one of two 2019 “Kitahonami” samples was positive (Figure 1). A phylogenetic analysis of the WYMV isolates, based on their RNA1 sequences, revealed that the obtained sequences belonged to genotype B, whose members are widely distributed in wheat-growing regions in northern Japan, including Hokkaido island (Ohki et al., 2014; Supplementary Figure S3A). In Japan, the WYMV isolates have been divided into three distinct pathotypes (I–III) based on their pathogenicity to wheat varieties, with pathotype II (genotype B) presenting in northern Japan, including Hokkaido island (Ohto et al., 2006). The WYMV isolate from the Naganuma field likely belongs to pathotype II, and symptoms could not be confirmed in the resistant wheat variety “Yumechikara,” in which a major quantitative trait loci, designated as *Q. Ymym*, against WYMV on chromosome 2D is known (Kobayashi et al., 2020; Figure 1; Supplementary Figure S3A and data not shown).

Two negative-sense RNA virus-like contigs (Wh18L_c253 and Wh19L_c20097) were identified from the pool-18L library (Table 2 and Supplementary Table S2). These two contig sequences are related to the NCMV (a planthopper-vectored rhabdovirus, genus *Cytorhabdovirus*, family *Rhabdoviridae*) and cover nearly the entire region of its genomic RNA (Supplementary Figure S2B). The concatenated contig sequence (Wh18L_c253/20097; deposited in DDBJ under Accession No., LC632071; Table 2) showed a high level of sequence identities with those of the Japanese (Accession No. AB030277) and Chinese (Hebei, GU985153) isolates of NCMV (98.5 and 93.8% nucleotide sequence identities, respectively). The distribution pattern of the mapped reads on the NCMV reference genome (4,186 mapped reads; Table 2) likely reflects the transcription gradient (a 3'–5' polar gradient of mRNA production), in which the transcripts of the N protein mRNA are the most abundant, with those of the downstream genes being at gradually lower levels (Dietzgen et al., 2017; Horie et al., 2021; Supplementary Figure S2B). The NJ tree, based on the L protein (RdRP), showed that the NCMV isolates are placed within a large sub-clade of cytorhabdoviruses (tentatively named subclade I), and formed a tight cluster together with other cereal rhabdoviruses, including barley yellow striate mosaic virus and maize yellow striate mosaic virus (Supplementary Figure S3B). Among the tested RNA samples in the pool-18L, NCMV was detected in one “Kitanokaori” sample (18_4-KTH; Figure 1). NCMV is known to be a viral agent of the stunted rosette symptom in wheat in East Asian countries (Tanno et al., 2000); however, the “Kitanokaori” plant (18_4-KTH) that was infected with the virus showed no stunting symptoms typical of the NCMV infection (data not shown). This may be due to the lower titer of NCMV in the host plants or other unknown reasons.

### Genome Analysis of a Novel Quinvirus and Detection of Its Strains

We identified 28 contigs in the two datasets (14 contigs each from the two sample pools, ranging from 203 to 3,828 nt) related to quinviruses (genera *Foveavirus* and *Carlavirus*, subfamily *Quinvirinae*) using the local BLAST analysis on the two datasets (Supplementary Table S2). Two concatenated sequences related to quinviruses were obtained by using the overlapped contigs, which share high nucleotide sequence identities at their overlapping regions with neighboring ones (Figure 2 and Supplementary Table S2). Subsequently, the entire genomic RNA region was identified by RT-PCR and Sanger sequencing using KTH-19-1 samples, following the 5' and 3' RACE analyses (Figure 2; Supplementary Table S1 and data not shown). The complete genomic sequences of the putative quinvirus were 8,412 and 8,411 nt in length, excluding the poly(A) tail; these were designated “strain a” and “strain b,” respectively (Table 2, Accession No., LC632066 and LC632067, respectively). In the 5' RACE clones, the GG (strain a, 9/16 clones) and G (strain b, 6/7 clones) nucleotides were mainly identified at the 5'-terminal ends of two genomic RNAs (data not shown). Both 5'-GG and 5'-G nucleotides at the 5'-terminal ends were most likely to be derived from 5'-end cap structure, as previously reported (Hirzmann et al., 1993; Kondo et al., 2014).

The genome organization of strain a was similar to that of foveaviruses and some others, with five ORFs, encoding replicase, TGB proteins, and CP, while strain b has an additional small ORF (ORF6) overlapping the CP gene (Figure 2). The presence of strain b with sequence variations within the 3'-terminal region were identified by the sequencing of the cloned 3' RACE products. The additional “A” residues presented at the potion 8,351 in some clones (UUAAAAA_8351_AnCCC…, additional A residues different from deposited one was underlined; of 12, 2, 1, and 1 3’-RACE clones had A_1_, A_4_, and A_5_; data not shown). These sequence variants lacked the extra ORF (ORF6) and it seems to be a minor population in the tested wheat sample (KTH-19-1), as the majority of the RACE clones (8/12), which would retain ORF6, had no additional A. The replicase of WVQ does not contain an alkylation B (AlkB)-like domain, which is commonly present in betaflexiviruses that infect perennial plants, such as fruit crops (Kondo et al., 2013; Moore and Meng, 2019; Figure 2). They showed moderate levels of nucleotide (79.5% for the entire genome) and amino acid sequence identities (68.6∼91.7%) to each other (Table 3). Since different species of quinviruses should have less than about 72% nt identity or 80% aa identity for quinviruses (King et al., 2011), these two sequences likely represent the strains of a single quinvirus species. It should be noted that there were other overlapping fragments (contigs 133 and 431 in pool-18L, marked with a white circle in Figure 2) with high nucleotide sequence identities (>97.0%) to the strain a reference sequence (Supplementary Table S2 and data not shown), suggesting that this strain consists of multiple variants.

**Table 3:** Comparison of predicted coding proteins between two WVQ strains.

Protein		Strain a	Strain b	Sequence identity^1^
Genome size		8,412 nt	8,411 nt	79.5%
ORF1	replicase	2069 aa (233.9 kDa)	2070 aa (233.9 kDa)	87.6%
ORF2	TGB1	223 aa (24.8 kDa)	223 aa (24.7 kDa)	86.6%
ORF3	TGB2	120 aa (13.4 kDa)	120 aa (13.4 kDa)	84.3%
ORF4	TGB3	85 aa (9.2 kDa)	85 aa (9.2 kDa)	68.6%
ORF5	CP	263 aa (28.6 kDa)	263 aa (28.6 kDa)	91.7%
ORF6	ORF6	–^2^	152 aa (17.3 kDa)^3^	–^2^

1 Using the NCBI BLAST-P-suite-2.

2 –: Not presented or no hits.

3 Some variants lack the ORF6 due to the insertion of A residue(s) at 8,351 nt (see text).

Along with these quinvirus sequences, some related contigs showed moderate levels of sequence similarity with the two strains (share 72.1–85.0% nucleotide sequence identity with the reference sequences), indicating the presence of a third strain (designated “strain c”) in the sample pools (Table 2; Supplementary Figure S2C; Supplementary Table S2 and data not shown). To confirm the presence of these three strains in the RNA samples, RT-PCR and subsequent direct Sanger sequencing were performed using strain-specific primer sets (Supplementary Table S1). A partial nucleotide sequence has been deposited as a representative of strain c sequence in DDBJ under Accession No., LC632068 (Table 2). All three strains were amplified in most of the tested samples in both pool-18L and pool-19L, although the amount of amplicons derived from strain c were relatively lower compared to those of the other two strains in some of the tested wheat plants (Figure 1). Unfortunately, the entire genomic sequence of strain c could not be determined *via* combinational RT-PCR and direct sequencing. Some overlapping contigs (contigs 936 and 1832 or 935 in pool-19L), likely derived from strain c, show high nucleotide sequence identities >95.9% to each other (Supplementary Table S2 and data not shown). Therefore, the mixed variant population of strain c likely existed in these pooled samples. This mixed population may have affected the direct sequencing analyses for the strain c. Further analyses are required to determine its entire genome.

### Taxonomical and Phylogenetic Analyses of WVQ

**Figure 3:**
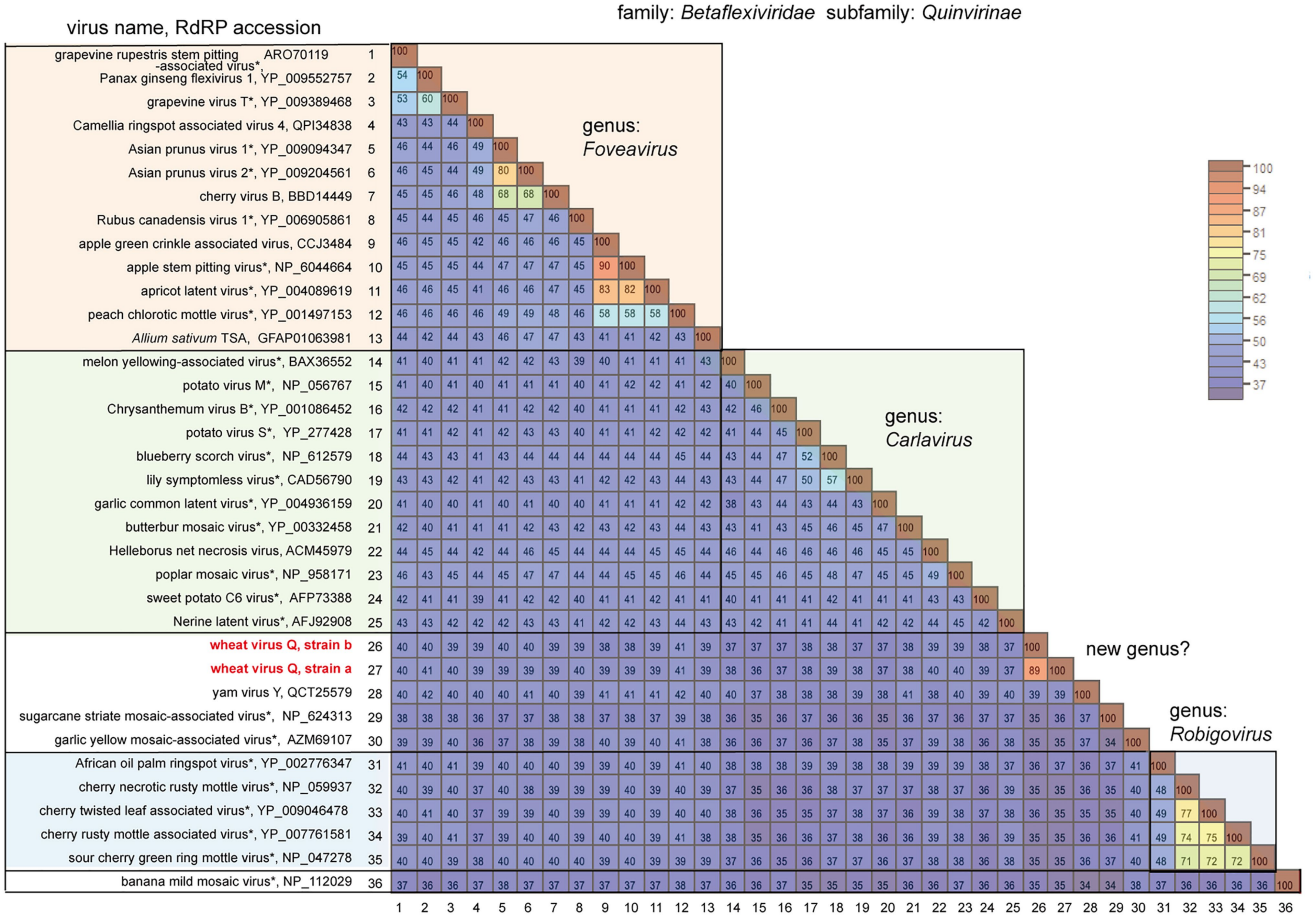
Pairwise comparison of the replicase (RdRP) encoded by quinviruses or their candidates. The results of pairwise comparisons are shown as a heatmap with each pairwise amino acid identity calculated using SDT ver. 1.2. The virus names with asterisks are representative members of the viral species.

BLAST-N analyses using the novel quinvirus sequences (strains a and b) as queries revealed that no significant genome-wide hits were detected (only for less than 12.0% for query coverage using other quinviruses; data not shown). PASC analysis of the genomic sequence of strain a showed 41.1% identity with that of a foveavirus – grapevine rupestris stem pitting-associated virus – which was the top hit sequence (Supplementary Figure S4A). Viruses among different genera in the *Quinvirinae* usually have less than about 45% nt identity in their genes (King et al., 2011), suggesting that WVQ represents a novel genus in the family. In the BLAST-P analyses, each predicted protein of the novel quinvirus (except for ORF6 in strain b) showed moderate levels of deduced amino-acid sequence identities (∼38.1–47.5%) with the corresponding proteins encoded by other quinviruses (mainly foveaviruses infecting fruit trees; Table 4). PASCs using STD also showed similar results (Figure 3 and Supplementary Figures S4B,C). Taken together, we propose that this newly discovered virus represents a virus species belonging to a novel genus in the family *Quinvirinae*.

**Table 4:** BLAST-P analyses using viral proteins of WVQ (strain a) as the queries.

Top hit virus	QC^1^	*E*-value	Identity	Accession
Query sequence: replicase				
Peach chlorotic mottle virus	85%	0.0	44.3%	AVD50414
Grapevine rupestris stem pitting-associated virus	84%	0.0	44.5%	QPB70005
Grapevine virus T	77%	0.0	47.9%	AYQ96168
Apple stem pitting virus	83%	0.0	42.7%	QKV49427
Panax ginseng flexivirus 1	75%		47.5%	YP_009552757
Query sequence: TGB1				
Rubus virus 1	99%	1e-53	44.0%	QLI58026
Apple stem pitting virus	97%	1e-49	43.8%	AGR66384
Peach chlorotic mottle virus	97%	6e-49	45.5%	YP_001497154
Apricot latent virus	97%	1e-48	44.2%	ADT91611
Apple green crinkle associated virus	97%	1e-48	43.3%	YP_006860590
Query sequence: TGB2				
Elderberry carlavirus A	87%	3e-26	47.2%	YP_009224930
Apple stem pitting virus	89%	1e-22	48.6%	AGR66384
Elderberry carlavirus B	89%	1e-22	44.4%	YP_009224936
Garlic common latent virus	89%	3e-21	46.9%	QED43133
Ilex cornuta carlavirus	89%	8e-21	45.0%	QJZ28443
Query sequence: TGB3				
No hits				
Query sequence: CP				
Peach chlorotic mottle virus	80%	3e-39	38.1%	AVD50418
Apple stem pitting virus	85%	2e-38	38.3%	AGR66254
Peach asteroid spot virus	71%	3e-38	40.1%	AAG48309
Garlic common latent virus	97%	1e-48	44.2%	QED43133
Ilex cornuta carlavirus	97%	1e-48	43.3%	QJZ28445

1 Query coverage (%).

**Figure 4:**
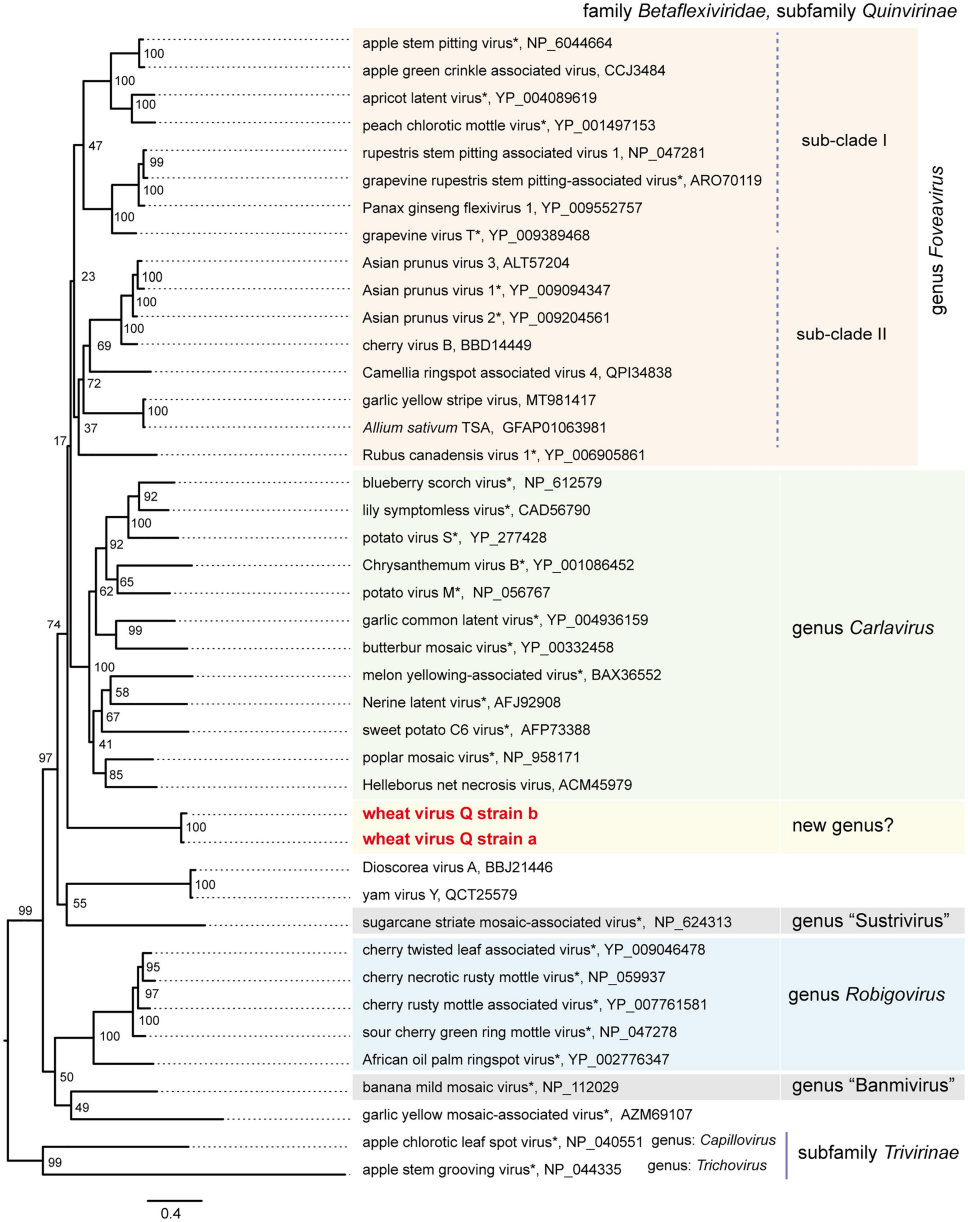
Phylogenetic relationships of the quinviruses and other unassigned related viruses. A maximum likelihood phylogenetic tree was constructed using a MAFFT alignment of the full-length replicase amino acid sequences. Ambiguously aligned sequences were removed using Gblocks with the stringency levels lowered for all parameters. A model LG + I + G + F was selected as a best-fit model for the alignment (the 1,235 positions remaining in the input dataset). The tree with the highest log likelihood (−43,188,80) is shown. The tree was drawn using the midpoint rooting method. The virus names referring to plant viruses [genera *Foveavirus*, *Carlavirus* (selected), *Robigovirus*, and floating (proposed genera “Banmivirus” and “Sustrivirus”) or unassigned members of subfamily *Quinvirinae*, family *Betaflexiviridae*] are followed by the GenBank accession or Ref-seq numbers of their sequences. Virus names with asterisks show representative members of the viral species. The two betaflexiviruses – apple chlorotic leaf spot virus (genus *Trichovirus*) and apple stem grooving virus (genus *Chapillovirus*) – of subfamily *Trivirinae* (family *Betaflexiviridae*) were used as the outgroups. The scale bar represents amino acid distances. The numbers at the nodes in the tree are bootstrap support values following 100 iterations.

To understand the phylogenetic relationships between WVQ and the known quinviruses, we constructed an ML tree using the amino-acid alignment of their entire replicase sequences. The constructed tree showed that the WVQ strains form a separate clade within the subfamily *Quinvirinae.* WVQ is distantly related to members of the three genera *Foveavirus*, *Carlavirus*, and *Robigovirus*, and other floating or unassigned quinviruses, such as SCSMaV (proposed genus “Sustrivirus”), banana mild mosaic virus (proposed genus “Banmivirus”), garlic yellow mosaic-associated virus, and yam virus Y (Gambley and Thomas, 2001; Thompson and Randles, 2001; Da Silva et al., 2019; Silva et al., 2019; Figure 4). Similar trends were also observed in the NJ trees based on CP and TGB, in which WVQ was placed separately from the three established genera (see also Ryu and Song, 2021; Supplementary Figures S5A,B).

### Small RNA Profiles of WYMV and WVQ

**Figure 5:**
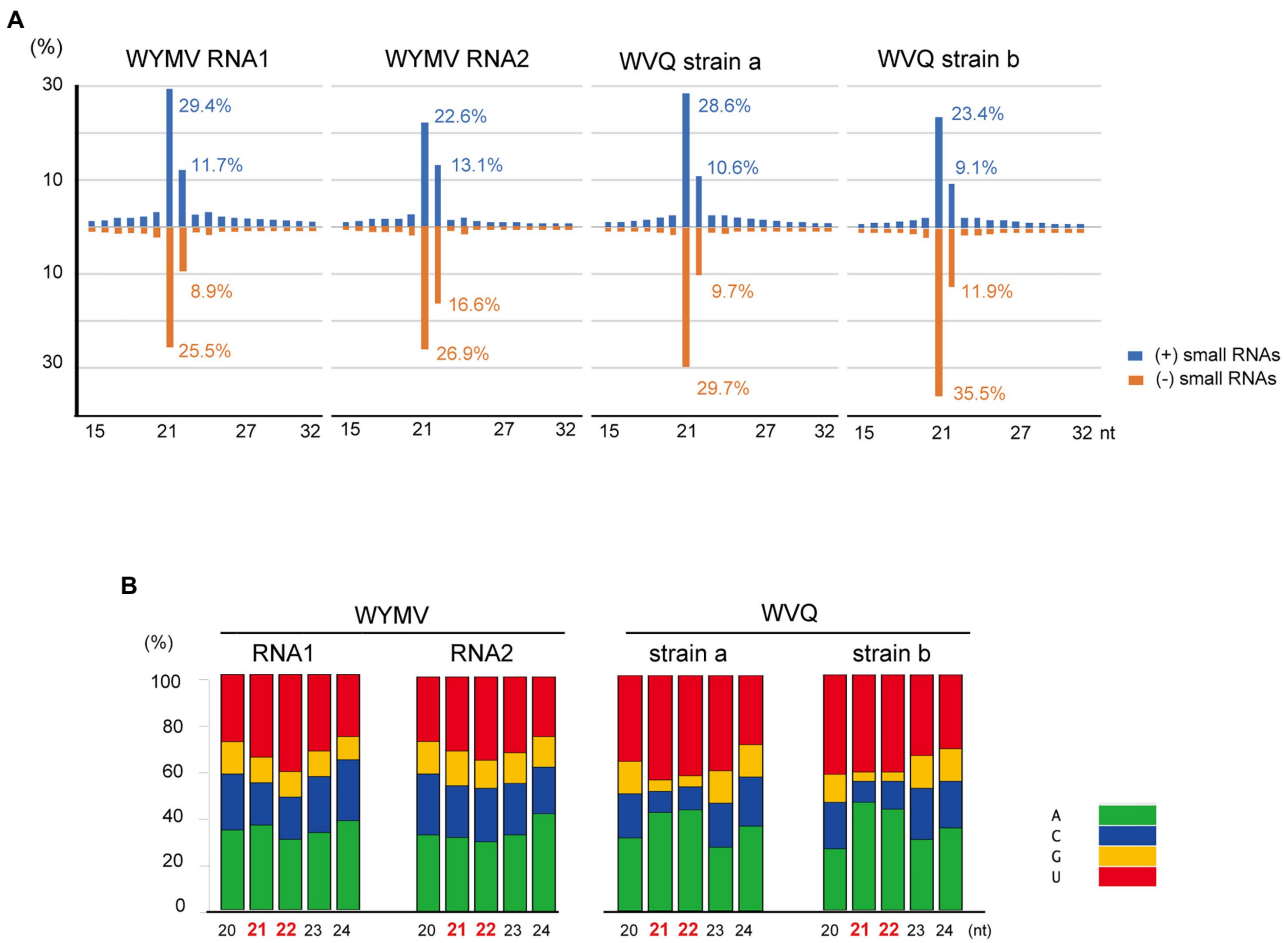
Viral-derived small RNA profiles of WYMV and WVQ in the wheat leaves (the pooled KTH-18-1 and −2 samples). **(A)** Proportion of plus (+)- and minus (−)-strand small RNAs sizes, 15–32 nt, are shown. **(B)** Proportions of 5ꞌ-terminal nucleotide sequences of viral-derived small RNAs in the wheat leaves. The composite bar graphs represent the percentage of the 5ꞌ-terminal nucleotide sequences for each size-class (20–24 nt). Other profiles of the viral-derived small RNAs are presented in Supplementary Figure S6.

Small RNA fractions of the wheat leaves (a pooled sample, KTH-18-1 and −2; Table 1) were deep sequenced to investigate the antiviral RNA silencing response. The small RNA reads (ranging 15–32 nt in length) mapped to the WYMV and WVQ were accounted for 0.5% (207,007 reads) and 0.2% (110,492 reads) of the total small RNAs, respectively. The small RNAs derived from both viruses were nearly equal positive and negative strands (45.9–59.6% for positive small RNA strands; Supplementary Figure S6A), predominantly 21 and 22 nt lengths for both strands (Figure 5A and Supplementary Figure S6B) and their 5'-terminal nucleotides were biased toward adenine (A) or uracil (U), which were particularly more prominent for 21 and 22 nt small RNAs in WVQ than those of WYMV (Figure 5B). Moreover, viral 21 and 22 nt small RNAs were distributed throughout the WYMV and WVQ genomes, with several distinct hotspots (Supplementary Figure S6C). Taken together, these analyses showed that WYMV- and WVQ-derived small RNAs possess the typical characteristics of viral small interfering RNAs (siRNAs; Li et al., 2017).

### Transmission of WVQ

**Figure 6:**
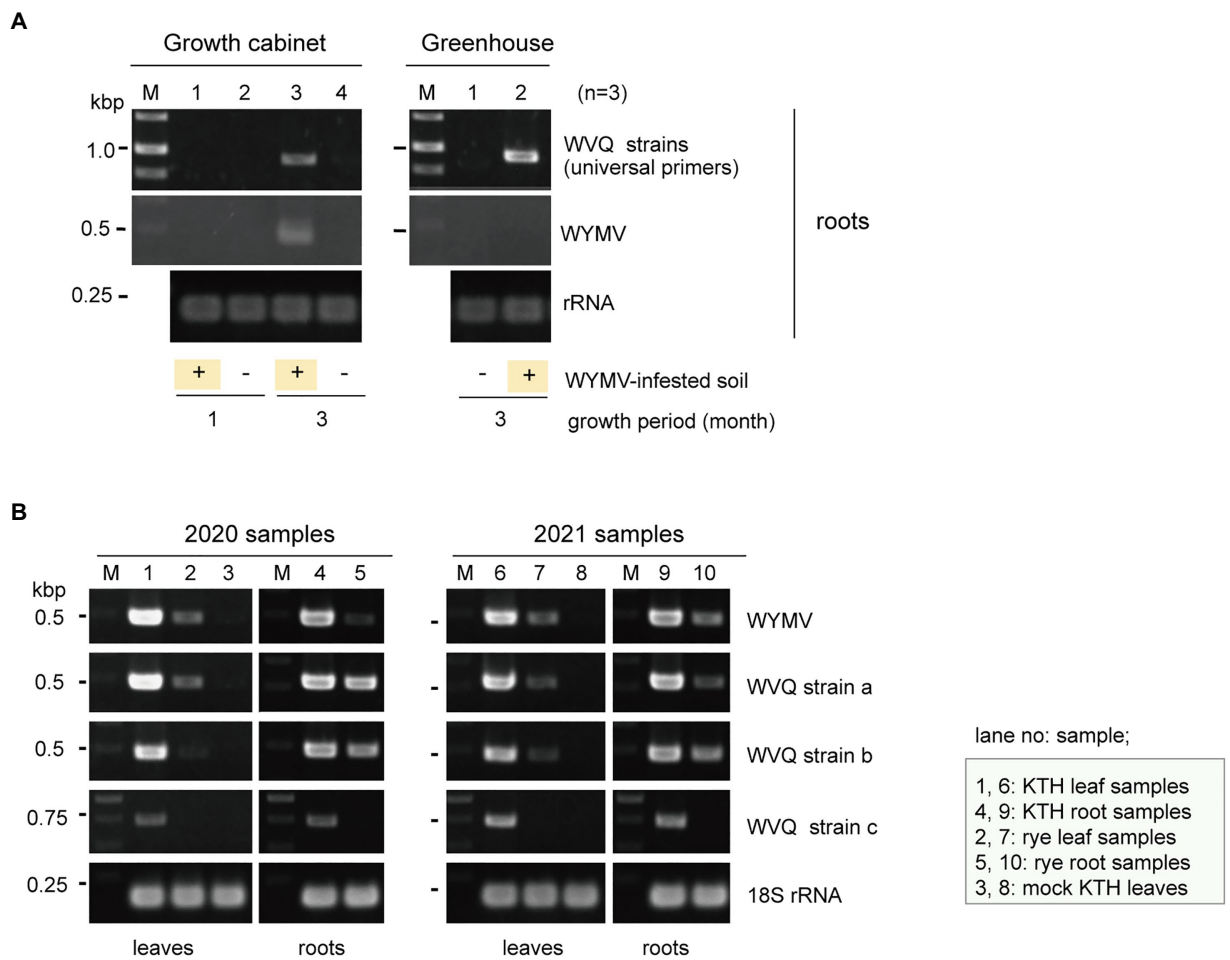
**(A)** Transmission assay of WVQ. RT-PCR detection of viruses in the roots of wheat plants planted in WVQ and wheat yellow mosaic virus (WYMV)-infested soil. Wheat grains were sown in pots containing the field soil and grown under the growth-cabinet condition (below 16°C) or the greenhouse condition (non-controlled temperature). Three pots for each treatment were used. Total RNA fractions from the root samples were pooled according to the treatment and subjected to RT-PCR analysis with a common primer set for the WVQ strains. **(B)** RT-PCR detection of WVQ and WYMV in leaves or roots of wheat or rye plants. Sample names and their respective tissue origin are shown below the panel or in the bottom right. Lanes 3 and 8 are RNA samples of mock-inoculated plants. KTH, cv. Kitahonami; rye, cv. Fuyumidori. The wheat 18S rRNA gene was used as an endogenous RNA reference. The primers used in the RT-PCR analyses are listed in Supplementary Table S1. The PCR products were analyzed in 1.5% agarose gel electrophoresis. The gel was stained with ethidium bromide and viewed under UV light. Selected PCR products were sequenced to confirm the amplification of the virus targets.

Our preliminary finding that WVQ was detected in WYMV-infected roots in the early spring (data not shown) when the temperature was relatively cool and thus the insects usually do not spread widely in the fields, suggests the possibility that WVQ may be transmitted to the roots through soil. Therefore, we aimed to examine the transmission of WVQ *via* soil under different conditions. Wheat seeds (cv. “Kitahonami”) were sown in pots containing soils from WYMV-infested fields or commercial soils as a control. One group of pots (three pots for each treatment) being kept under growth-cabinet control at approximately 16°C, which is generally allowed for WYMV infection, while the other group of pots was kept in the greenhouse (non-controlled temperature), where WYMV infection is usually more difficult (Zhang et al., 2021). Under the growth cabinet condition, both WVQ and WYMV were detected by RT-PCR in the roots of wheat after 3 months of growth, but not after 1 month (Figure 6A). After 3 months of growth in the greenhouse, WVQ, but not WYMV, was detected in the roots of the plants (Figure 6A). No systemic infection or virus symptoms were observed in the aerial parts of the plants in either trial (data not shown). Thus, it was revealed that WVQ is potentially soil-transmissible, and this virus may require lower temperature and/or other unknown conditions for systemic infection but not for root infection unlike WYMV.

### WVQ Infection in Rye Plants

WVQ strains were constantly detected by RT-PCR in the roots and leaves of the wheat plants (cv. “Kitahonami”) that were grown in WYMV-infested fields at Naganuma over 4 consecutive years (2018–2021; Table 1; Figures 1, 6B and data not shown). To investigate the occurrence of WVQ in other crops, the rye samples were also collected from the same field in 2020 and 2021 and were subjected to RT-PCR. The results showed that WVQ strains (except for the strain c) were also detected in both leaves and roots of rye plants along with co-infecting WYMV (Figure 6B), indicating that WVQ is able to infect rye plants.

## Discussion

The occurrence of wheat yellow mosaic disease in East Asian countries has been predominantly associated with bymovirus infection, but it is possible that the magnitude of disease severity is also influenced by the viral communities in the wheat fields that sustain this disease. In this study, we investigated the wheat leaf virome associated with yellow mosaic disease in Hokkaido, Japan (Table 1). We identified three viruses – WYMV (a bymovirus), NCMV (a cytorhabdovirus), and a novel quinvirus (WVQ, at least three strains) – from two pooled samples obtained in 2 consecutive years (Table 2). In addition to the plant virus sequences, mycovirus-like contigs (at least 43) were also found in the two datasets (data not shown). Although the leaf meta-RNA-seq may be able to provide information on the mycoviral communities in crop-associated fungal populations (Al Rwahnih et al., 2011; Marzano and Domier, 2016; Chiapello et al., 2020; Ma et al., 2021), many of the obtained contigs were only partial genomic sequences. Thus, further analyses are required to determine their entire genomes.

In this study, we have identified a novel quinvirus (family *Betaflexiviridae*), tentatively named “wheat virus Q.” Among the three WVQ strains identified in the Naganuma field, two strains were determined on their complete genomic sequences (Tables 2, 3; Figure 2 and Supplementary Figure S2). The sequence and phylogenetic analyses support the taxonomic status of WVQ as a distant evolutionary lineage (a member of the new genus) in the *Quinvirinae*, but the phylogenetic trees also show low statistical support for a deep node (for replicases) or formed two or three distinct clades (for CP and TGB1) of foveaviruses (Table 4; Figures 3, 4; Supplementary Figures S4, S5). Thus, more as-yet-unreported members of the foveaviruses and other quinviruses, including WVQ-related viruses, are needed in order to obtain more accurate phylogenetic relationships for these taxonomic studies. The three WVQ strains (a, b, and c) were simultaneously detected in most of the tested samples (Figure 1 and Table 1). In addition, the RNA-seq data suggest the presence of WVQ variants belonging to each reference strains (at least strains a and c; Figure 2; Supplementary Figure S2C and Supplementary Table S2). Together, these indicate that WVQ infection in nature commonly forms a mixed population derived from multiple strains and variants. It is still unclear how these WVQ variants form mutant clouds (viral quasispecies) in the wheat plants (Domingo et al., 2012; Domingo and Perales, 2019) The biological significant of a mixed population for the WVQ infection is worth investigating in the future. The presence of WYMV and WVQ siRNAs in wheat leaves suggests that infection of both viruses induces antiviral RNA silencing responses in the field conditions (Figure 5 and Supplementary Figure S6). Previous report showed that WYMV siRNAs have different characteristics between leaves and roots (Li et al., 2017). Because like WYMV, WVQ also potentially infects plant through roots, it is interesting to examine whether WVQ siRNAs also have differential characteristics between leaves and roots.

WVQ is the second quinvirus found to infect cereal crops following SCSMaV, which is distributed across a limited area in Queensland, Australia (Choi et al., 1999; Thompson and Randles, 2001). WVQ co-existed with WYMV in at least four different wheat-producing areas in the Hokkaido (NY, unpublished results), suggesting its widespread occurrence in this island. In addition, some unannotated short sequence fragments related to WVQ strains (mostly its 3'-terminal parts) were identified in the wheat EST libraries (cultivar “Chinese Spring”; Supplementary Figure S7), whose source plant materials were grown in Japan but outside of Hokkaido island (Kawaura et al., 2005; Mochida et al., 2006), suggesting the possibility that WVQ may also occurs in other islands in Japan. The natural infection by WVQ has also been found in the rye plants in the same Naganuma field (Figure 6B). WYMV is transmitted to wheat by the soil-borne vector *P. graminis* (Tamada and Kondo, 2013) and the field soil used for transmission experiment was infested with *P. graminis* (HK, unpublished results), while the underground mechanism, or possible biological vector, of WVQ transmission is still unknown (Figure 6A). Interestingly, as well as WYMV can be detected in nucleic acid fractions extracted from soil taken from an area in the Naganuma field where no wheat planting has taken place for the last 1 year (NY, unpublished results). This may suggests that WVQ is associated with unknown vector as for bymoviruses associated with *P. graminis* in the soil or present in a stable and/or transmissible form in the soil similarly to tobamoviruses (Ikegashira et al., 2004). It seems that SCSMaV is also soil transmissible *via* an unknown mechanism (Hughes, 1961; Thompson and Randles, 2001). Only natural vectors of some carlaviruses have been reported (Ryu and Song, 2021), while the vector of other members of subfamily *Quinvirinae* is still unknown. There is no particular sequence feature specifically present in WVQ and SCSMaV and absent in other members of the *Quinvirinae* (except for the cysteine-rich protein of carlaviruses).

WVQ was present in at least four consecutive growing seasons in the wheat plants coinfected with WYMV (Table 1; Figures 1, 6B), and were also detected in different wheat-producing areas in Hokkaido as mentioned above, revealing the common coexistence of WVQ and WYMV in wheat fields in Hokkaido. This high incidence of co-infection by the two unrelated viruses is reminiscent of a soil-borne pathosystem, the lettuce big-vein disease (BVD). BVD has been associated with two unrelated negative-sense RNA viruses, Mirafiori lettuce big-vein virus (MLBVV, an ophiovirus in the family *Aspiviridae*) and lettuce big-vein associated virus (LBVaV, a varicosavirus in the family *Rhabdoviridae*), transmitted by a root-infecting fungus *Olpidium brassicae* (Kormelink et al., 2011; Maccarone, 2013). MLBVV is responsible to the induction of BVD symptoms, but LBVaV alone never shows symptoms (Lot et al., 2002; Roggero et al., 2003). The co-infection by these two viruses was frequently observed in the lettuce fields; thus, yet unknown interactions between two soil-borne viruses may play pivotal roles in this pathosystem. Therefore, the impact of WVQ infection on WYMV-induced yellow mosaic disease, particularly on symptom development and grain production, is worthy of detailed investigations. It will also be a future challenge to determine whether there is a trans enhancement in accumulation between the two wheat viruses.

## Data Availability Statement

The virus and virus-like sequences derived from this study have been submitted to the GenBank/DDBJ/ENA with the accession numbers LC632066–LC632071.

## Author Contributions

HK designed the experiments. NY and TT collected the samples. HK, NY, MF, KM, KH, HH, and NS performed the experimental work and discussion. HK analyzed the data and wrote the draft manuscript. lBA, NY, TT, and NS were involved in manuscript revision. All authors have given their approval to the final version of the manuscript.

## Conflict of Interest

The authors declare that the research was conducted in the absence of any commercial or financial relationships that could be construed as a potential conflict of interest.

## Publisher’s Note

All claims expressed in this article are solely those of the authors and do not necessarily represent those of their affiliated organizations, or those of the publisher, the editors and the reviewers. Any product that may be evaluated in this article, or claim that may be made by its manufacturer, is not guaranteed or endorsed by the publisher.

## Footnotes

^1^ https://www.ncbi.nlm.nih.gov

^2^ https://nucleobytes.com/enzymex/index.html

^3^ http://nucleobytes.com/index.php/4peaks

^4^ https://www.ncbi.nlm.nih.gov/Structure/cdd/wrpsb.cgi

^5^ https://www.ncbi.nlm.nih.gov/sutils/pasc/viridty.cgi

^6^ http://mafft.cbrc.jp/alignment/server/

^7^ http://molevol.cmima.csic.es/castresana/Gblocks_server.html

^8^ http://www.atgc-montpellier.fr/phyml-sms/

^9^ http://tree.bio.ed.ac.uk/software/
