# Supplementary material for: Identification of a Novel Quinvirus in the Family Betaflexiviridae That Infects Winter Wheat
Source: Front Microbiol. 2021 Aug 19;12:715545. doi: 10.3389/fmicb.2021.715545 (PMC8417474; doi:10.3389/fmicb.2021.715545)
Supplement: Supplementary file 1 [file Data_Sheet_1.PDF]

Supplementary Data

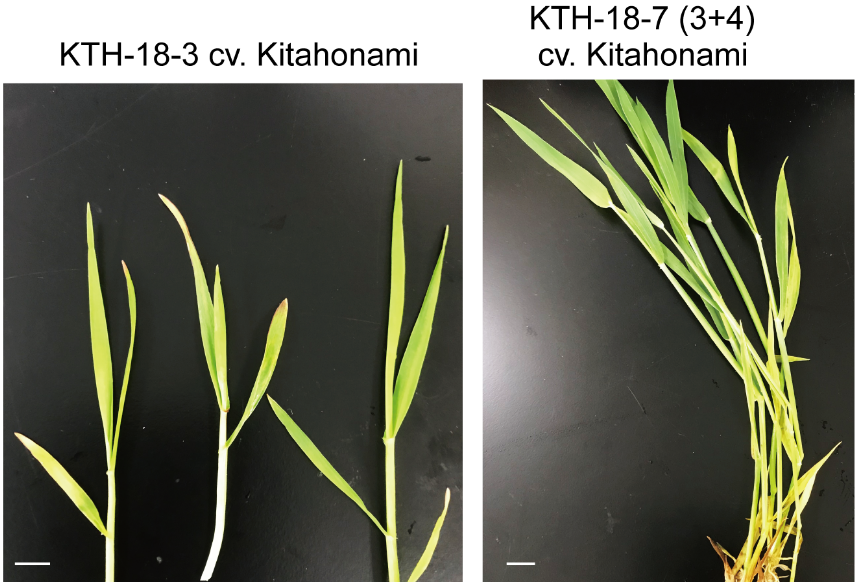

FIGURE S1 | Representative wheat-leaf samples, showing yellow mosaic symptoms (KTH-18-3, KTH-18-7; see Table 1), that were used for RNA-seq analysis. The white bars equal 2 cm.

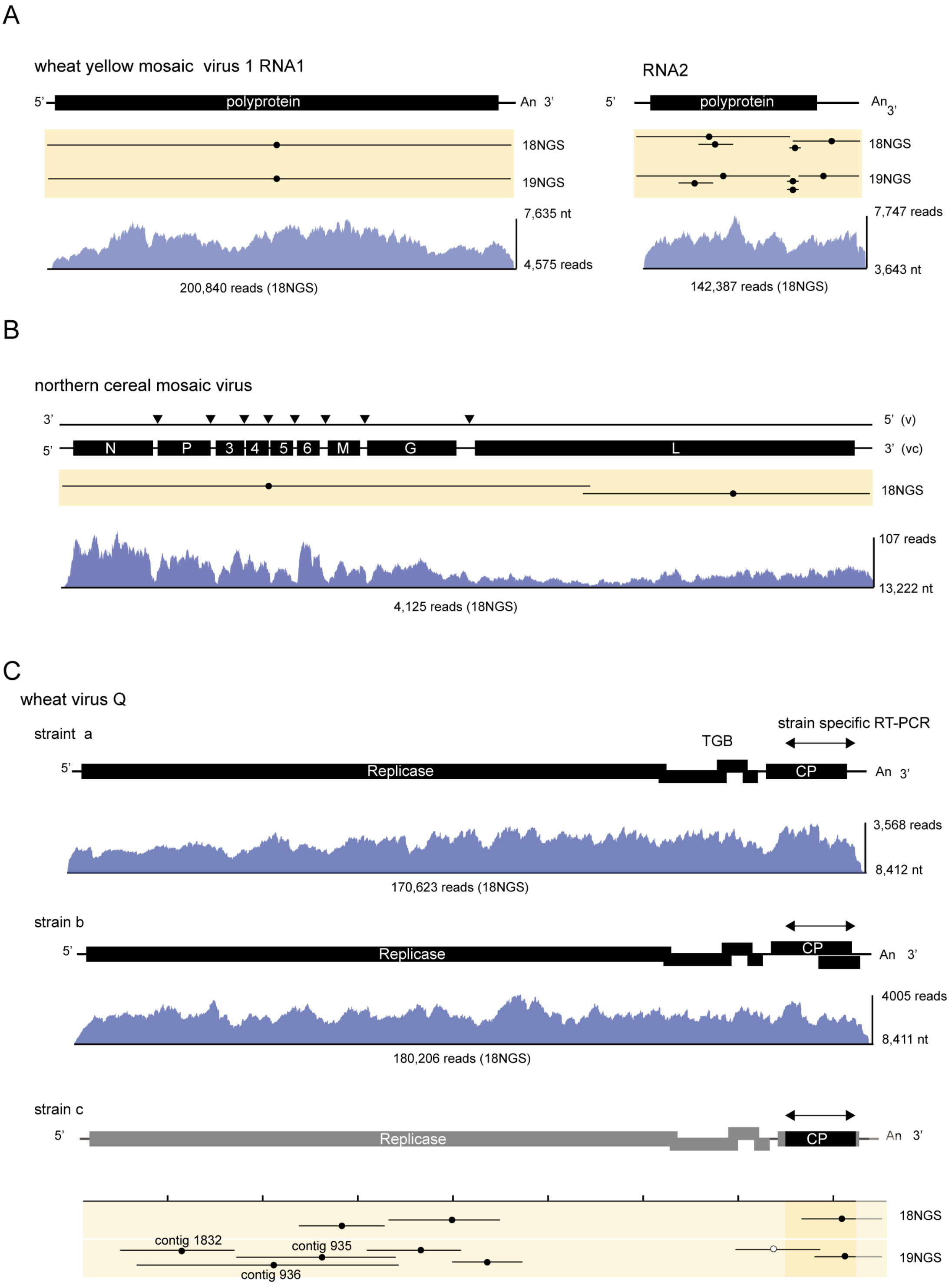

FIGURE S2 | Read-depth coverage throughout the genomes virus sequences identified from the RNA-seq analyses of wheat leaves. (A) wheat yellow mosaic virus (WYMV, a bymovirus, the isolate Nagamuma-1). (B) Northern cereal mosaic virus (NCMV, a cytorhabdovirus, the isolate Nagamuma-2). v: genomic

sequence (negative sense); vc: complimentary (positive sense). (C) WVQ (a novel quinvirus) strains a, b and c were shown. The black boxes in the genomic RNA show open reading frames (ORFs). Virus-sequence contigs of stain c from two separate total RNA pools of wheat-leaf samples (18NGS: pool-18L; 19NGS: pool-19L) are shown as thin black lines. The lines marked with a black circle were the contigs listed in Supplementary Table S2, while the line marked with an open circle was used for a sequence construction for the CP and 3'-UTR regions. The read depth coverages of the data from pool-18L via the CLC Genomics Workbench version 11 using via the CLC Genomics Workbench using the Read Mapping algorithm with default parameters (match score=1; mismatch cost=2; length fraction=0.5; similarity fraction=0.8) are shown. The Y-axis shows the mapping-read coverage with the maximum read number.

A

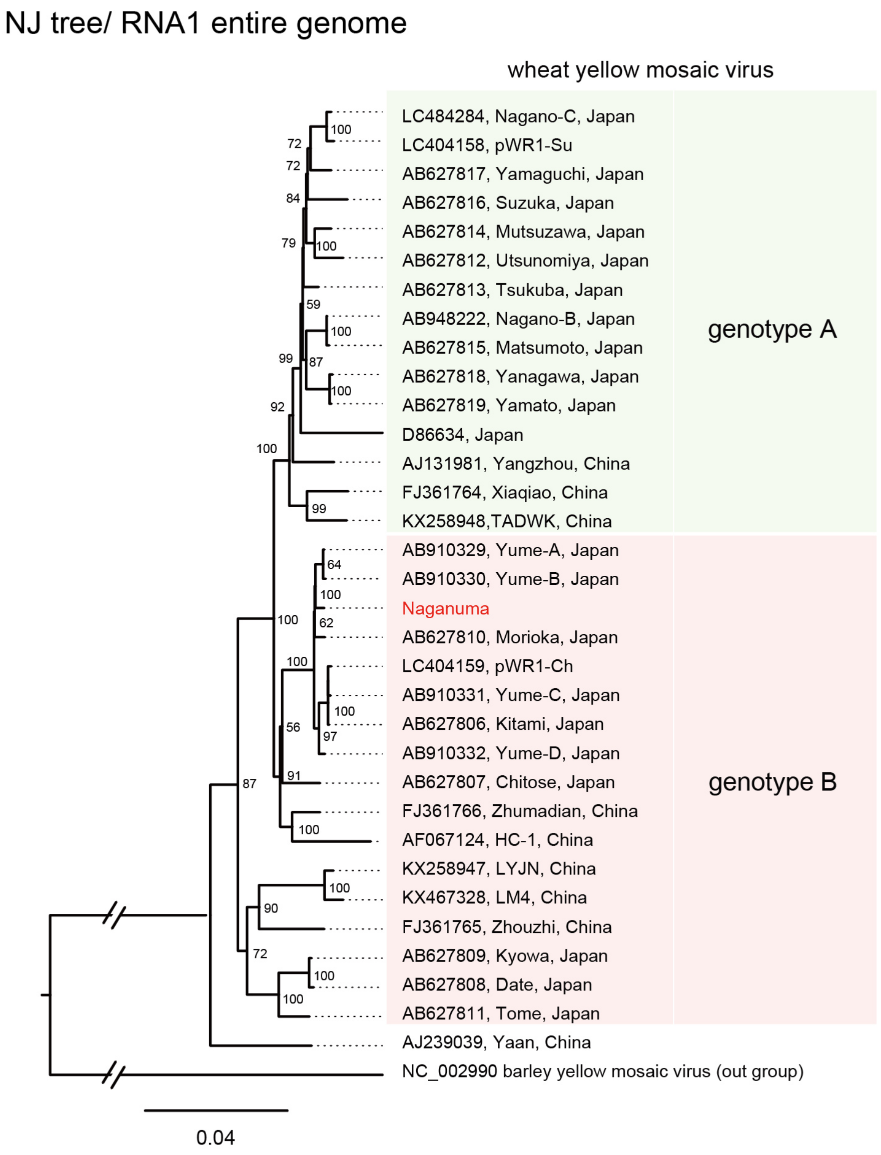

FIGURE S3 | Phylogenetic relationships of WYMV **(A)** and NCMV **(B).** NJ tree based on the RNA1 sequences of WYMV isolates (A) or plant rhabdovirus L protein amino acid sequences (B) via MAFFT. Ambiguously aligned sequences were removed using Gblocks with the stringency levels lowered for all parameters. The barely yellow mosaic virus (genus *Bymovirus,* family *Potyvirudae*) and lettuce big vein-associated virus (genus *Varicosavirus*, family *Rhabdoviridae*) were used as the outgroup in A and B, respectively. The virus names are followed by the GenBank accession or Ref-seq numbers of their sequences. The virus names with asterisks show representative members of the plant rhabdovirus species in B shown a representative member of the viral species following their GenBank accession or Ref-seq numbers. The scale bar represents distances. The numbers at the nodes in the tree are bootstrap support values following 1,000 iterations.

Figure S3 continued

B

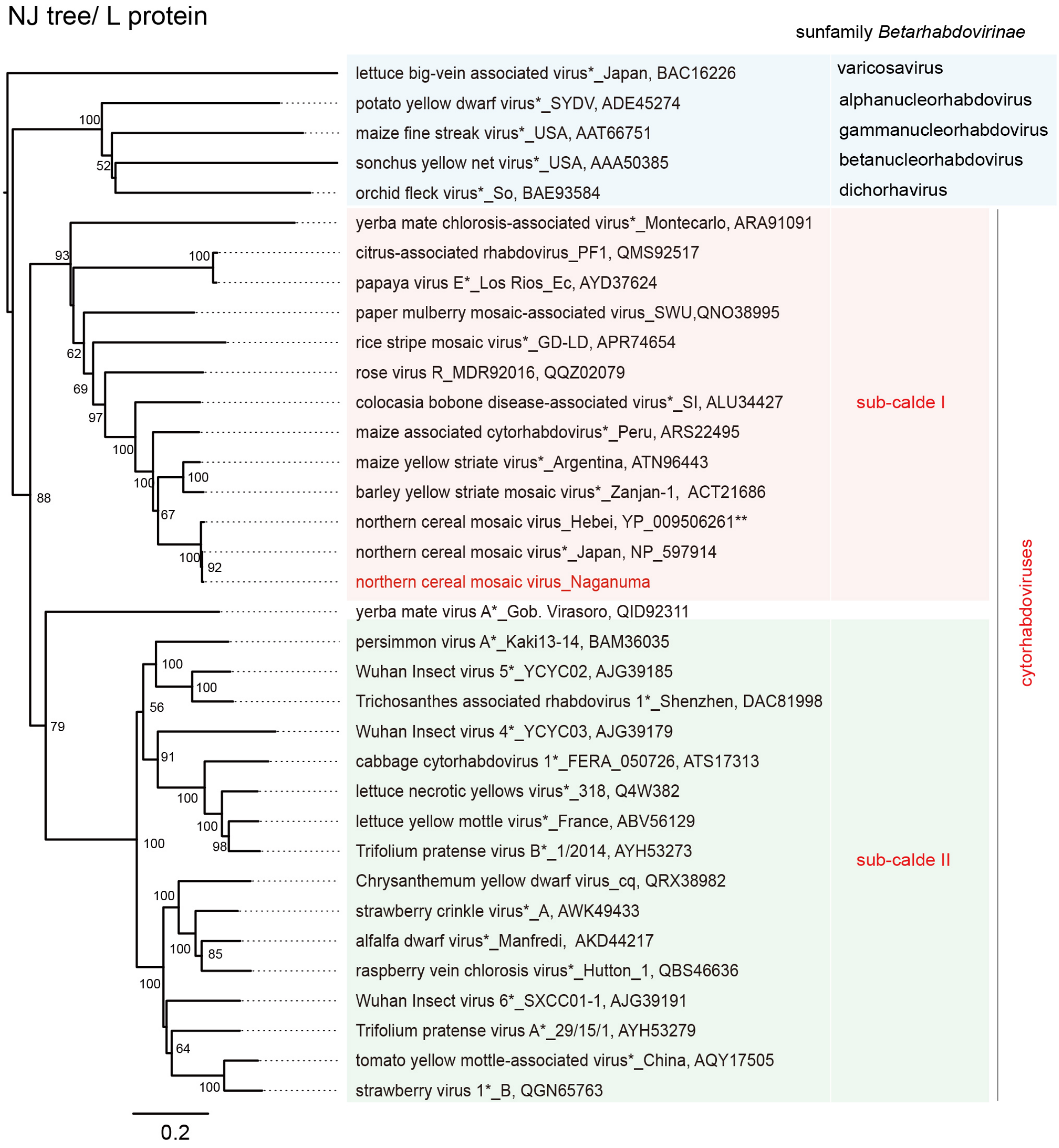

A

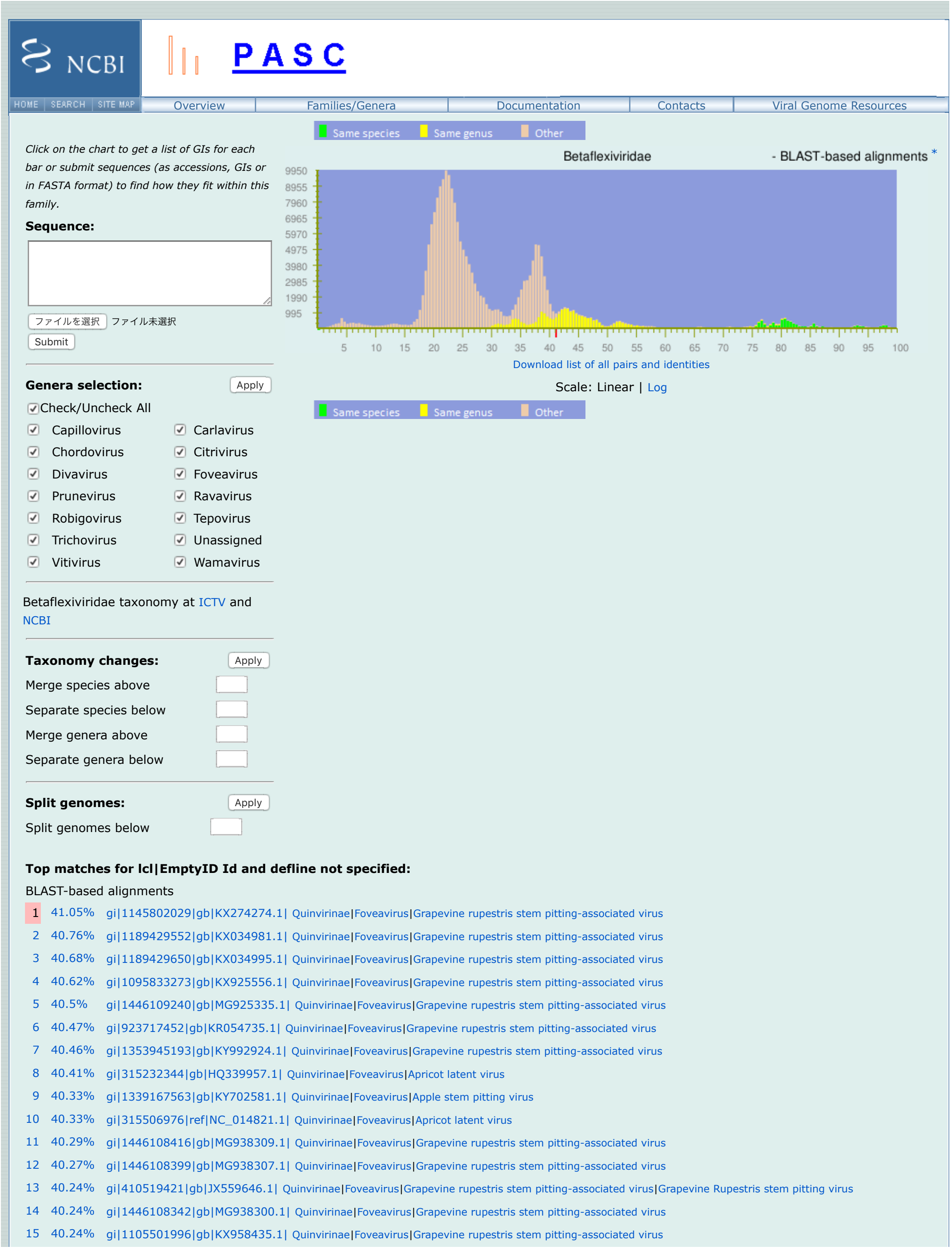

FIGURE S4 | The result of PASC analysis, showing the frequency distribution (the top right) and BLAST results (the bottom) from the complete genome sequence comparison of betaflexiviruses with WVQ as a query. **(B and C)** Pairwise comparison of the coat proteins (B) and TGB1 proteins (C) encoded by quinviruses or their candidates. The results of pairwise comparisons are shown as a heatmap with each pairwise amino acid identity calculated using SDT ver. 1.2.

Figure S4 continued

B

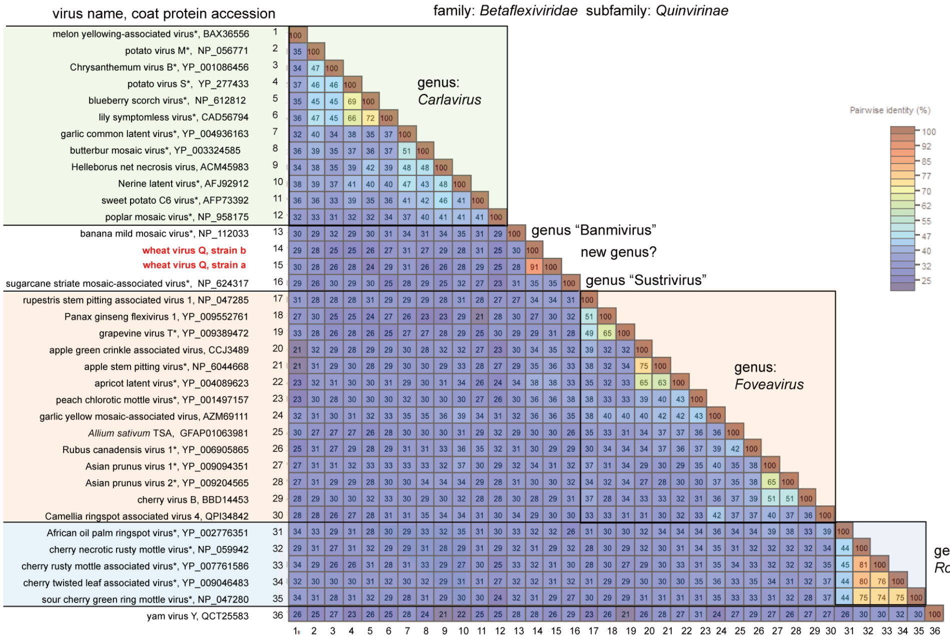

C

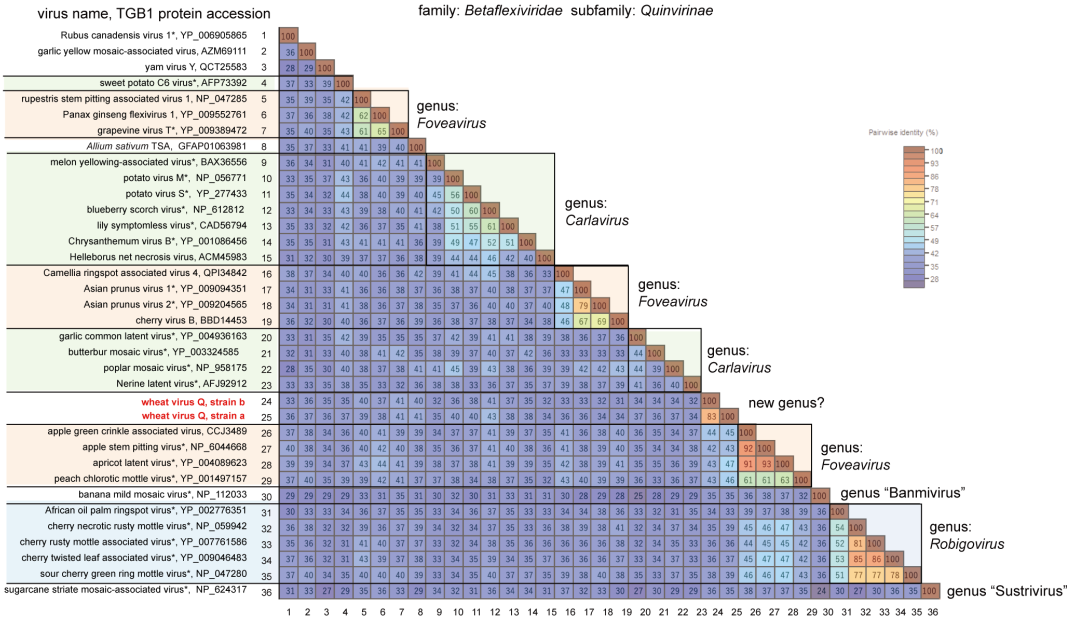

A

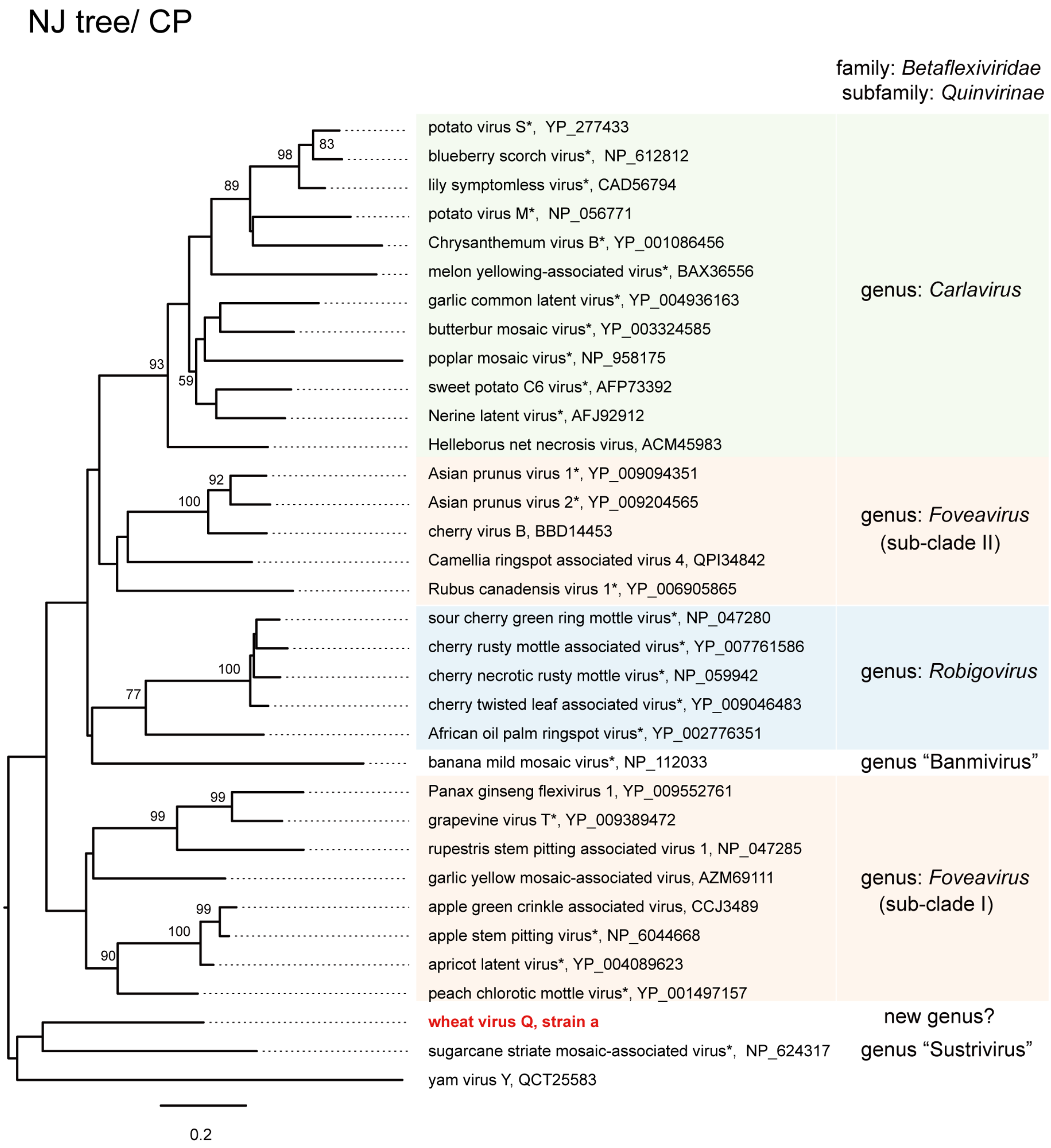

FIGURE S5 | Phylogenetic relationships of the quinviruses and other unassigned related viruses based on the CP **(A)** and TGB protein 1 (TGB1) **(B)**. NJ tree based on the CP (A) or TGB1 (B) amino acid sequences via MAFFT. The scale bar represents distances. Ambiguously aligned sequences were removed using Gblocks with the stringency levels lowered for all parameters. The virus names referring to plant viruses (genera *Foveavirus, Carlavirus, Robigovirus,* and floating (proposed genera "Banmivirus" and "Sustrivirus") or unassigned members of subfamily *Quinvirinae*, family *Betaflexiviridae*) are followed by the GenBank accession or Ref-seq numbers of their sequences. Virus names with asterisks are representative members of the viral species. The tree was drawn using the midpoint rooting method. The numbers at the nodes in the tree are bootstrap support values following 1000 iterations.

Figure S5 continued

B

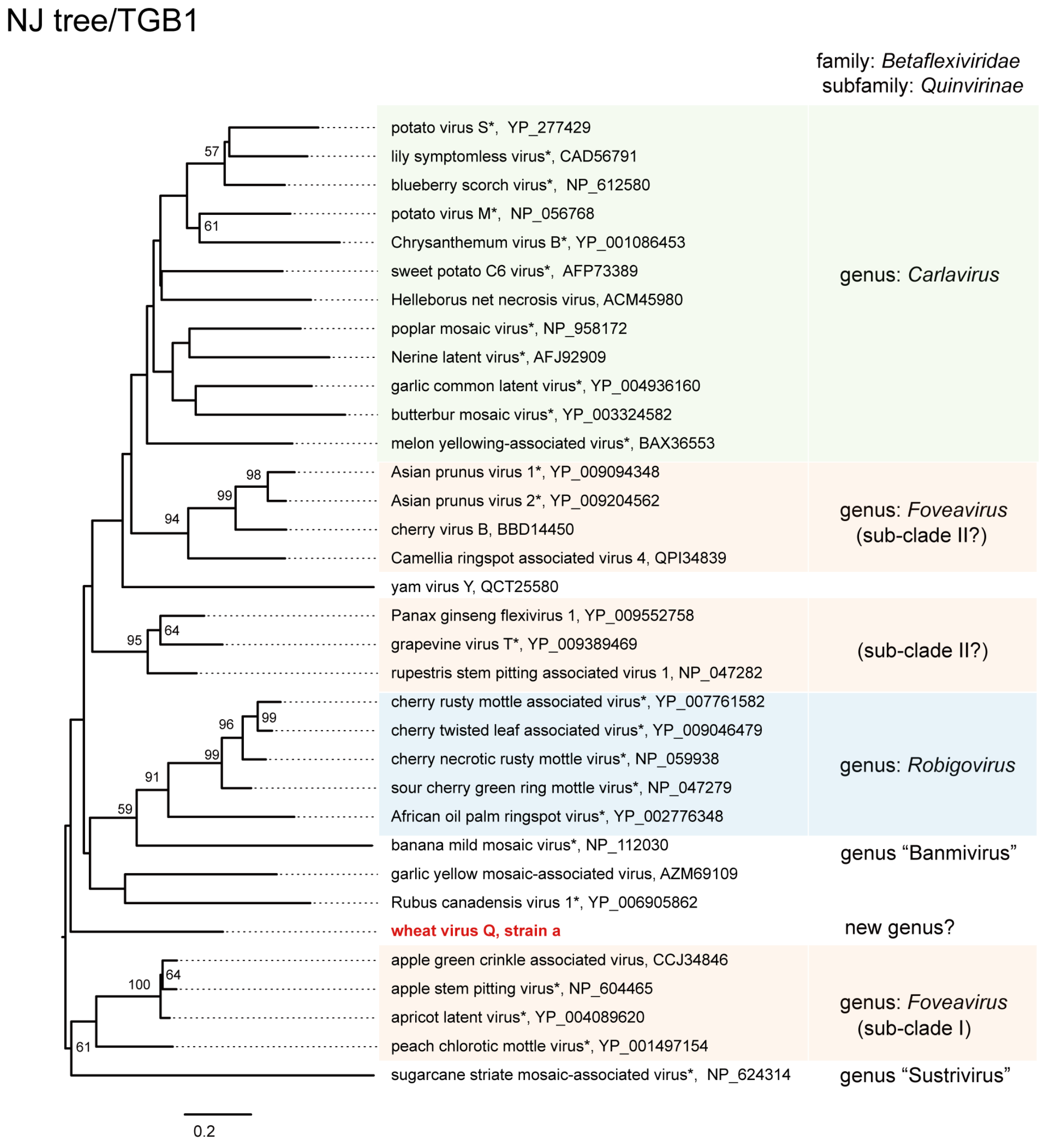

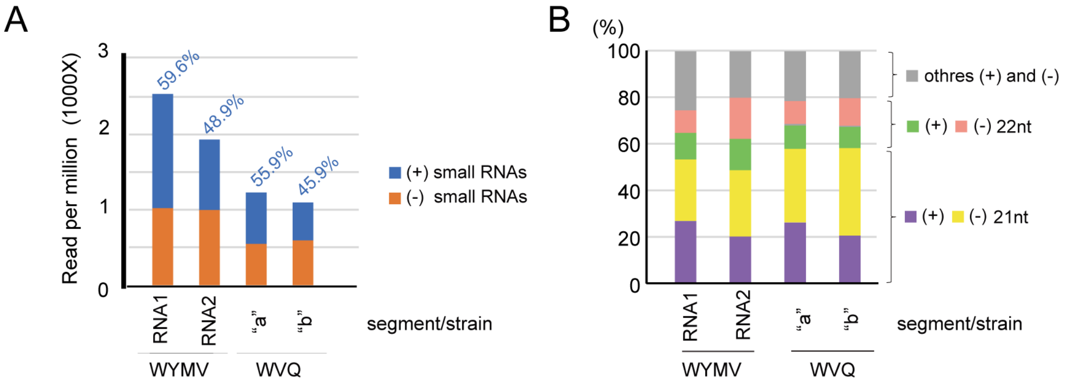

FIGURE S6 | Viral-derived small RNA profiles of WYMV and WVQ in the wheat leaves (the pooled KTH-18-1 and -2 samples). **(A, B)** Proportion of plus (+)- and minus (-)-strand small RNA sizes, 15 to 32 nt (A) or 21 and 22 nt (B). **(C)** Distribution of viral-derived small RNAs (15–30 nt) along the genomes of WYMV (RNAs 1 and 2) and WVQ (strains a and b). The 21 and 22 nt small RNAs were plotted using MISIS. The bars above (blue) and below (red) the axis represent plus- and minus-strand reads, respectively.

Figure S6 continued

C

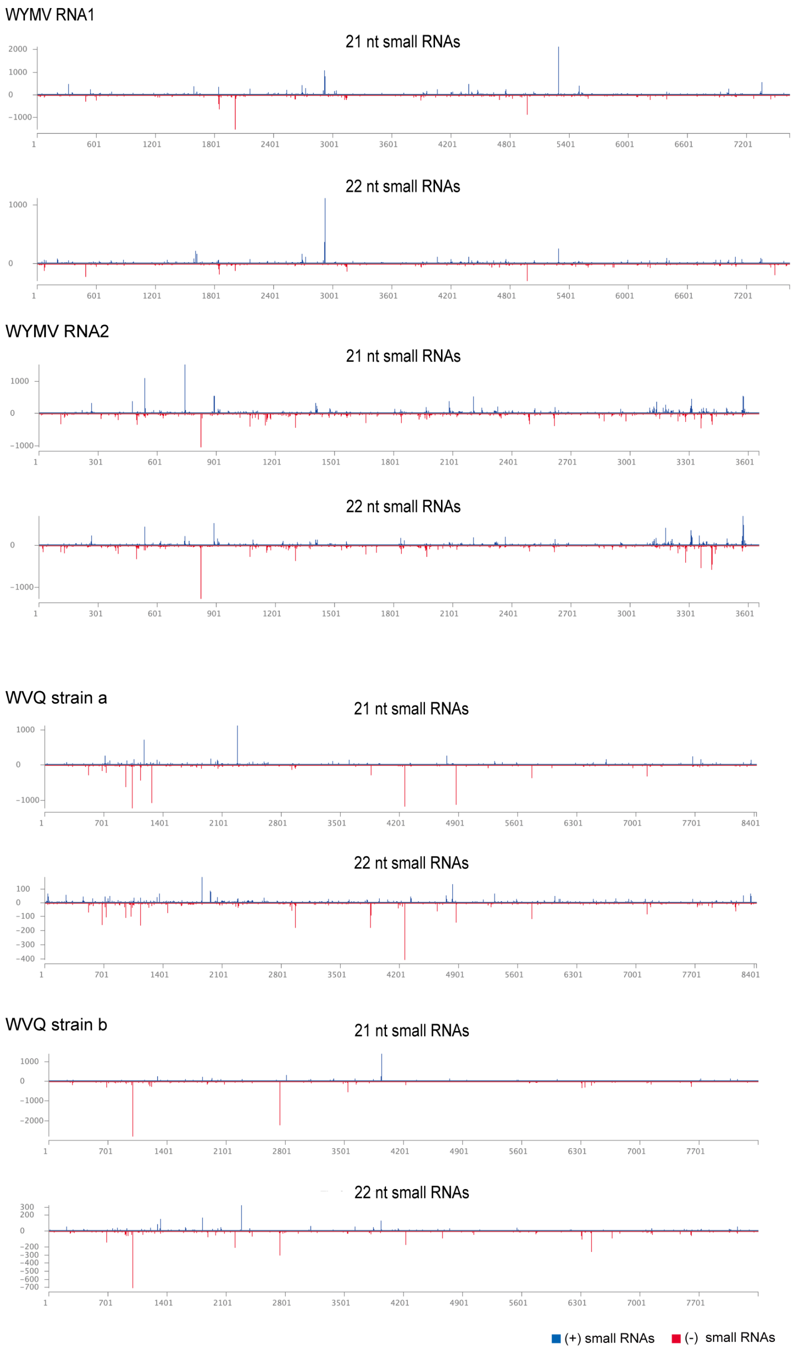

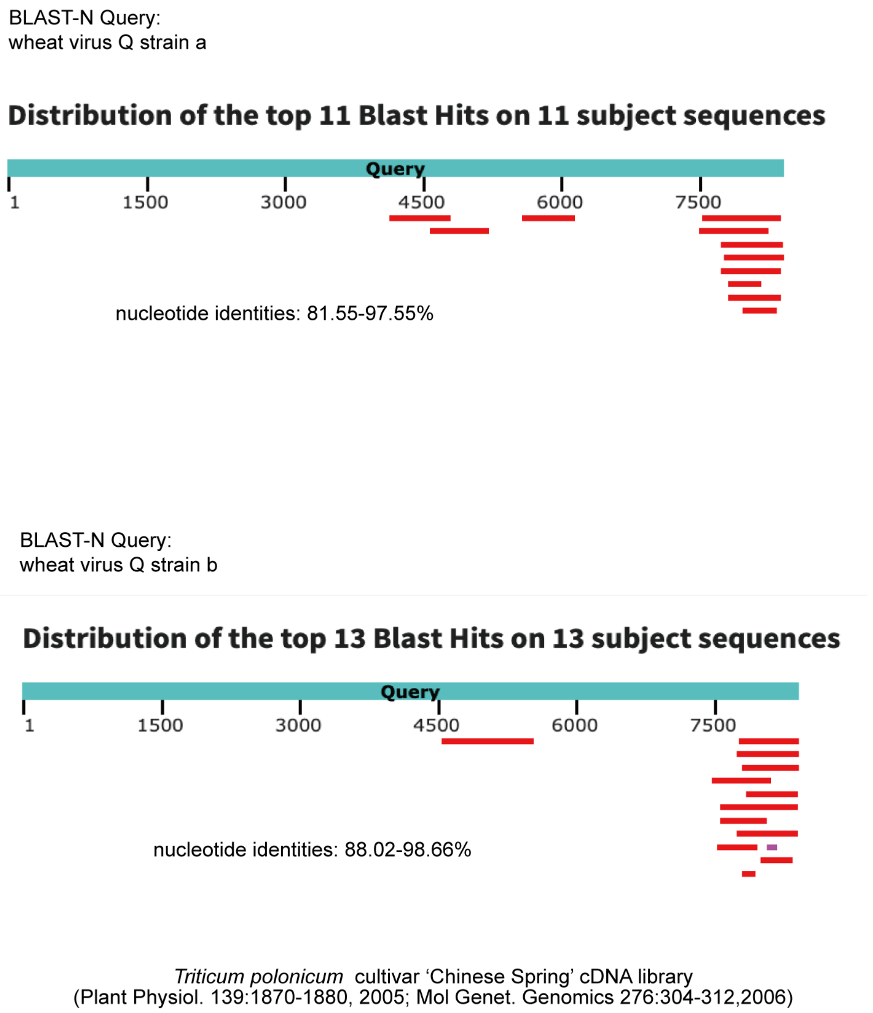

FIGURE S7 | Graphical summaries of the BLAST-N results using two WVQ strains as queries. Fragments of WVQ-related sequences were identified from the wheat EST datasets. Some wheat EST hits were overlapped in the BLAST-N results of both strains. A wheat cDNA (No. AK330490, 'Chinese Spring') related to WVQ strain b was also identified from the database during BLAST-N analysis.

TABLE S1 | List of primers (selected) used in this study.

name	5'–3'
**RT-PCR**	
WYMV-RNA1_F	CGCGCAATCGACCTAATATACGACG
WYMV-RNA1_R	CAAGTAAGAGTGCAGAACGCCGCCC
NCMV-NC_F	TTCAAAGACTATATTCCTCAGGGAG
NCMV-NC_R	TCTCTAGCATCCCGTTCCACCCATC
WVQ-a_F	GAGGTAGCCGAGGCCTTCACTACAA
WVQ-a_R	GGTTTATTCATTATTATTCAGGCAG
WVQ-b_F	ACCCATAACTGCTATTGTGGATGTC
WVQ-b_R	TCCCAATTCGCAGGAGGGAAGTCA
WVQ-c_F	GCCCATAACTTCTGTTGTGGACACG
WVQ-c_R	TTTTATTTACTTACCTCAACCTTCT
WVQ-uni_F	CATGCTTTAGATCCAGAGGATGCTG
WVQ-uni_R	CGACAGGTATAACGCTCACCTGTAG
18SrRNA_F	GTGACGGGTGACGGAGAATT
18SrRNA_R	GACACTAATGCGCCCGGTAT
**5'RACE**	
5'-end phosphorylated primer	
WVQ-R-5PHO	5'P-AGCTCATCATGAAGAAAGAGATTGG
5'RACE-PCR for strains a and b	
WVQ-a_R1	ATCGAAAAGAAAATAATTCTCTAAA
WVQ-a_F1	TATCGAAGCGATAAACACCCTCGTC
WVQ-b_R1	TCAAAGAGGAAAAAGTTCTCGAGG
WVQ-b_F1	TATCGAGGCAATCAATACTCTCGTC
**3'RACE**	
1st strand cDNA synthesis	
Anchored dT-NotI*	AAGAATTCGCGGCCGCAGGAATTTTTTTTTTTTTTTTT
3'RACE-PCR for strains a and b	
Not I-anchor*	GGAAGAATTCGCGGCCGCAGGA
WVQ-a_F	see above
WVQ-b_F	see above
**WVQ genome sequencing****	
WVQ-a_1abF	AAGTCTCAGGATGGCCACTCTTCGG
WVQ-a_1aR	GGAATGTCAACAGCAGCGTCTTCCT
WVQ-a_2aF	TCCAAACTCCTGCAATTTCTGAAGA
WVQ-a_2aR	CTCGACGCAAGGCTCTAAAATTTCA
WVQ-a_3aF	GATCCTCAAGGATAAGGACTACAAT
WVQ-a_3aR	ACCAAAGCGGTGAGATCTTCGAGAT
WVQ-a_4aF	GGAGACAAGAGGTTGTTGAGAGGGAC
WVQ-a_R	see above
WVQ-b_1abF	see above
WVQ-b_1bR	ATATCAACAGAAGTTTCATGACAAG
WVQ-b_2bF	TCCCTGCTCTTTCTCCAGTCGTTCA
WVQ-b_2bR	ATTCGGTGAAGGTTCAAGAATGTCC
WVQ-b_3bF	AGCCCTGGGACTTTTCTTGACAGGC
WVQ-b_3bR	TCTGAAAGCAAGTGGCAAGTATTGG
WVQ-b_4bF	AAAACAAGGGTCTTTGGCCAAGGGG
WVQ-b_R	see above

*: replaced with primers that were supplied by the 3′-Full RACE Core Set (TaKaRa Bio).

**: see Fig. 2.

TABLE S2 | List of plant-virus-like contigs from the wheat-leaf RNA-seq analysis and the results of their BLAST-P analyses.

Contig name	Consen sus length	Total read count	Average coverage	results of the local BLAST-N*			results of the local BlastX (query contigswere larger than 1.0 kb)*			
				Lowest E-value	Accession	Description	Lowest E-value	Accession	Description	
**POOL-18**										
Wheat18L_1_(paired)_contig_88_mapping	7425	195853	2621.7	0	NC_002350	Wheat yellow mosaic virus RNA 1, complete sequence	0	NC_002350	Wheat yellow mosaic virus RNA 1, complete sequence	WYMV
Wheat18L_1_(paired)_contig_38_mapping	2521	96907	3861.3	0	NC_002349	Wheat yellow mosaic virus RNA 2, complete sequence	0	NC_002349	Wheat yellow mosaic virus RNA 2, complete sequence	WYMV
Wheat18L_1_(paired)_contig_1462_mapping	1159	41471	3590.7	0	NC_002349	Wheat yellow mosaic virus RNA 2, complete sequence	0	NC_002349	Wheat yellow mosaic virus RNA 2, complete sequence	WYMV
Wheat18L_1_(paired)_contig_5330_mapping	511	2944	577.04	0	NC_002349	Wheat yellow mosaic virus RNA 2, complete sequence				WYMV
Wheat18L_1_(paired)_contig_1970_mapping	230	1057	445.91	1E-101	NC_002349	Wheat yellow mosaic virus RNA 2, complete sequence				WYMV
Wheat18L_1_(paired)_contig_253_mapping	8590	3215	37.701	0	NC_002251	Northern cereal mosaic virus, complete genome	0	NC_002251	Northern cereal mosaic virus, complete genome	NCMV
Wheat18L_1_(paired)_contig_20097_mapping	4669	1001	21.618	0	NC_002251	Northern cereal mosaic virus, complete genome	0	NC_002251	Northern cereal mosaic virus, complete genome	NCMV
Wheat18L_1_(paired)_contig_340_mapping	3038	65599	2148.2	3E-67	NC_018448	Sweet potato virus C-6, complete genome	0	NC_029085	Elderberry carlavirus A isolate EBCVA, complete genome	WVQ-b
Wheat18L_1_(paired)_contig_5192_mapping	1482	18739	1178.5	3E-69	NC_029086	Elderberry carlavirus B isolate EBCVB, complete genome	3E-126	NC_003499	Blueberry scorch virus, complete genome	WVQ-c
Wheat18L_1_(paired)_contig_431_mapping	1166	11604	936.39	1E-47	NC_035203	Grapevine virus T isolate Cho, complete cds	8E-138	NC_035203	Grapevine virus T isolate Cho, complete cds	WVQ-a
Wheat18L_1_(paired)_contig_134_mapping	2280	28005	1206.6	0.0006	NC_014821	Apricot latent virus, complete genome	1E-56	NC_009892	Peach chlorotic mottle virus, complete genome	WVQ-a
Wheat18L_1_(paired)_contig_133_mapping	2216	15622	672.7	0.0076	NC_029088	Elderberry carlavirus D isolate EBCVD, complete genome	2E-43	NC_009892	Peach chlorotic mottle virus, complete genome	WVQ-a
Wheat18L_1_(paired)_contig_2354_mapping	2181	53562	2352	4E-06	NC_012038	Helleborus net necrosis virus, complete genome	1E-149	NC_009892	Peach chlorotic mottle virus, complete genome	WVQ-a
Wheat18L_1_(paired)_contig_187_mapping	1051	11203	960.77	1E-08	NC_014821	Apricot latent virus, complete genome	8E-32	NC_040643	Panax ginseng flexivirus 1 isolate Changbai, complete genome	WVQ-c
Wheat18L_1_(paired)_contig_72_mapping	1291	27647	2135.5	0.0044	NC_028868	Asian prunus virus 2 isolate Bungo Q-1256-01, complete genome	2E-40	NC_038324	Melon yellowing-associated virus, isolate: M22	WVQ-b
Wheat18L_1_(paired)_contig_459_mapping	910	13575	1302.9	2E-12	NC_018714	Apple green crinkle associated virus complete genome				WVQ-a
Wheat18L_1_(paired)_contig_175_mapping	352	4666	1166	2E-26	NC_018714	Apple green crinkle associated virus complete genome,				WVQ-a
Wheat18L_1_(paired)_contig_1858_mapping	732	15346	1930	3E-09	NC_029087	Elderberry carlavirus C isolate EBCVC, complete genome				WVQ-a
Wheat18L_1_(paired)_contig_3765_mapping	215	2703	1159.9	3E-27	NC_029088	Elderberry carlavirus D isolate EBCVD, complete genome				WVQ-a
Wheat18L_1_(paired)_contig_607_mapping	753	19477	2536	4E-07	NC_029089	Elderberry carlavirus E isolate EBCVE, complete genome				WVQ-c
Wheat18L_1_(paired)_contig_8936_mapping	306	1235	406.01	4E-46	NC_011525	Potato latent virus, complete genome				WVQ-c
**POOL-19**										
Wheat19L_1_(paired)_contig_362_mapping	7702	111603	1459.6	0	NC_002350	Wheat yellow mosaic virus RNA 1, complete sequence	0	NC_002350	Wheat yellow mosaic virus RNA 1, complete sequence	WYMV
Wheat19L_1_(paired)_contig_740_mapping	2583	44650	1735.4	0	NC_002349	Wheat yellow mosaic virus RNA 2, complete sequence	0	NC_002349	Wheat yellow mosaic virus RNA 2, complete sequence	WYMV
Wheat19L_1_(paired)_contig_2864_mapping	1004	15849	1567.1	0	NC_002349	Wheat yellow mosaic virus RNA 2, complete sequence	0	NC_002349	Wheat yellow mosaic virus RNA 2, complete sequence	WYMV
Wheat19L_1_(paired)_contig_1065_mapping	561	2284	361.76	0	NC_002349	Wheat yellow mosaic virus RNA 2, complete sequence				WYMV
Wheat19L_1_(paired)_contig_2865_mapping	230	2608	1078.8	1E-102	NC_002349	Wheat yellow mosaic virus RNA 2, complete sequence				WYMV
Wheat19L_1_(paired)_contig_2866_mapping	230	736	305.1	1E-101	NC_002349	Wheat yellow mosaic virus RNA 2, complete sequence				WYMV
Wheat19L_1_(paired)_contig_778_mapping	1591	64297	3887	1E-80	NC_013527	Butterbur mosaic virus, complete genome	7E-153	NC_029086	Elderberry carlavirus B isolate EBCVB, complete genome	WVQ-a
Wheat19L_1_(paired)_contig_936_mapping	2723	17095	628.76	6E-05	NC_028975	Asian prunus virus 3 isolate Nanjing, complete genome	2E-104	NC_009892	Peach chlorotic mottle virus, complete genome	WVQ-c
Wheat19L_1_(paired)_contig_267_mapping	1855	59347	3075.2	5E-11	NC_029088	Elderberry carlavirus D isolate EBCVD, complete genome	5E-85	NC_009892	Peach chlorotic mottle virus, complete genome	WVQ-a
Wheat19L_1_(paired)_contig_935_mapping	1661	17162	1033	0.8423	NC_023423	Pithovirus sibericum isolate P1084-T, complete genome	5E-35	NC_009892	Peach chlorotic mottle virus, complete genome	WVQ-c
Wheat19L_1_(paired)_contig_97_mapping	1219	40971	3283.1	0.0003	NC_014821	Apricot latent virus, complete genome	7E-43	NC_009892	Peach chlorotic mottle virus, complete genome	WVQ-a
Wheat19L_1_(paired)_contig_1832_mapping	1196	14756	1218.7	2E-06	NC_024449	Cherry twisted leaf associated virus isolate CTLV_8431	1E-81	NC_009892	Peach chlorotic mottle virus, complete genome	WVQ-c
Wheat19L_1_(paired)_contig_9864_mapping	1109	9724	845.35	0.0131	NC_003870	Sugarcane striate mosaic-associated virus, complete genome	5E-47	NC_009892	Peach chlorotic mottle virus, complete genome	WVQ-b
Wheat19L_1_(paired)_contig_360_mapping	2166	19399	850.16	0.0074	NC_025388	Asian prunus virus 1 isolate tatao5, complete genome	1E-77	NC_006550	Sweet potato chlorotic fleck virus, complete genome	WVQ-b
Wheat19L_1_(paired)_contig_2529_mapping	3828	38239	970.07	1E-66	NC_029086	Elderberry carlavirus B isolate EBCVB, complete genome	0	NC_019025	Rubus canadensis virus 1 isolate BM-01, complete genome	WVQ-b
Wheat19L_1_(paired)_contig_248_mapping	1040	34812	3248.2	4E-09	NC_029087	Elderberry carlavirus C isolate EBCVC, complete genome	2E-59	NC_001948	Rupestris stem pitting associated virus-1, complete genome	WVQ-a
Wheat19L_1_(paired)_contig_2463_mapping	686	15323	2175	5E-05	NC_029087	Elderberry carlavirus C isolate EBCVC, complete genome				WVQ-c
Wheat19L_1_(paired)_contig_787_mapping	580	10021	1688.5	9E-46	NC_031089	Ligustrum virus A isolate SK, complete genome				WVQ-c
Wheat19L_1_(paired)_contig_10922_mapping	309	2001	589.15	2E-43	NC_029089	Elderberry carlavirus E isolate EBCVE, complete genome				WVQ-c
Wheat19L_1_(paired)_contig_10924_mapping	203	342	163.51	2E-29	NC_029089	Elderberry carlavirus E isolate EBCVE, complete genome				WVQ-c

*: against NCBI viral RefSeq collection
